# Immunoglobulin A Nephropathy: Molecular Pathogenesis and Targeted Therapy

**DOI:** 10.1002/mco2.70382

**Published:** 2025-09-08

**Authors:** Xu‐Jie Zhou

**Affiliations:** ^1^ Renal Division Kidney Genetics Center Key Laboratory of Renal Disease National Health Commission Peking University First Hospital Peking University Institute of Nephrology Peking University Institute of Nephrology Key Laboratory of Chronic Kidney Disease Prevention and Treatment (Peking University) Ministry of Education, and State Key Laboratory of Vascular Homeostasis and Remodeling Peking University Beijing China

**Keywords:** B lymphocyte, clinical trials, complement, IgA nephropathy, molecular pathogenesis, targeted therapy

## Abstract

Immunoglobulin A nephropathy (IgAN), the most prevalent primary glomerulonephritis globally, is characterized by mesangial IgA deposition and heterogeneous clinical trajectories. Historically, management relied on renin–angiotensin system inhibition and empirical immunosuppression, yet high lifetime kidney failure risk persists despite optimized care. This review synthesizes advances in molecular pathogenesis, highlighting how the traditional multi‐hit hypothesis—while foundational for targeted therapy development—fails to capture IgAN's recurrent, self‐amplifying nature. We introduce the “spiral hypothesis” as a dynamic model of cyclical immune‐injury cascades, better explaining disease chronicity and necessitating sustained maintenance therapy. Emerging targeted therapies—including B‐cell targeted agents (e.g., APRIL/BAFF inhibitors), complement inhibitors (e.g., iptacopan), and mucosal immunomodulators (e.g., TRF‐budesonide)—enable early intervention addressing both upstream immunological drivers and downstream fibrotic pathways. We critically evaluate treat‐to‐target frameworks, defining remission endpoints (proteinuria <0.3 g/day, hematuria resolution, estimated glomerular filtration rate slope <−1 mL/min/year) and emphasizing biomarker‐guided personalization. The paradigm shift toward proactive management prioritizes individualized therapeutic sequencing of novel agents based on dynamic risk stratification. Future priorities include optimizing protocols for high‐risk phenotypes and refining long‐term safety monitoring to ensure sustainable efficacy.

## Graphical abstract

The future of IgAN treatment likely involves combining several therapies to target multiple disease mechanisms simultaneously. Clinical trials are ongoing to evaluate the efficacy and safety of various combinations. As our understanding of IgAN pathogenesis evolves, more targeted and personalized treatment strategies are expected to emerge.

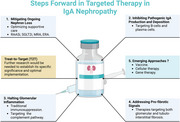

## 1 Introduction

Immunoglobulin A (IgA) nephropathy (IgAN), first described by Berger and Hinglais in 1968 [1, 2, 3], remains the most common primary glomerulonephritis worldwide, with a striking geographic disparity in prevalence, accounting for up to 40% of glomerular diseases in Asia but only 10–20% in Western populations [4, 5, 6, 7, 8, 9, 10, 11, 12, 13, 14, 15, 16, 17]. Characterized by mesangial deposition of galactose‐deficient IgA1 (Gd–IgA1) immune complexes, IgAN progresses to end‐stage kidney disease (ESKD) in 20–40% of patients within two decades, despite optimized supportive care [18, 19]. Historically considered benign, recent longitudinal cohorts challenge this view, demonstrating a median kidney failure‐free survival of only 11–12 years even among patients with subnephrotic proteinuria [18, 19]. The disease's molecular pathogenesis, rooted in a “multi‐hit” cascade of mucosal immune dysregulation, autoantibody production, and complement activation, has only recently been unraveled, paving the way for mechanism‐driven therapies [8, 9, 14].

The past decade has witnessed a paradigm shift in IgAN management, marked by United States Food and Drug Administration (US FDA) approvals of targeted therapies including targeted‐release formulation budesonide (TRF‐budesonide) (2023), complement inhibitors (iptacopan, accelerated approval 2024), and endothelin (ET) receptor antagonists (ERAs) (sparsentan 2023, atrasentan 2025), alongside emerging strategies such as BAFF (B‐cell activating factor, also known as BLyS, TALL‐1, CD257, or TNFSF13B) and APRIL (A Proliferation‐Inducing Ligand, also known as CD256 or TNFSF13) inhibitors undergoing phase 3 evaluation (sibeprenlimab, telitacicept), and cellular therapies in preclinical exploration [20].

However, critical challenges persist: (1) Existing therapies like corticosteroids show limited efficacy in high‐risk patients and carry significant safety risks; (2) Renin–angiotensin system inhibitors (RASi), while foundational, fail to halt progression in 20–40% of cases; (3) Novel agents face accuracy limitations in biomarker‐guided patient selection and long‐term safety uncertainties. These gaps underscore three key research questions: (1) How can we optimize risk stratification to identify patients needing early aggressive therapy? (2) What is the optimal sequencing of supportive care, immunosuppression, and targeted agents? (3) Can we develop cost‐effective strategies for global accessibility? This review navigates these controversies—including immunosuppression timing, corticosteroid roles, and therapy integration—amidst an increasingly complex therapeutic landscape, as clinicians now face the challenge of navigating an increasingly complex therapeutic landscape while balancing efficacy, safety, and cost effectiveness.

To ensure methodological transparency, this review employed a systematic literature search strategy. We searched PubMed and Cochrane Library databases (2000–2025) using the following terms: (“IgA nephropathy” OR “IgAN”) AND (“pathogenesis” OR “targeted therapy” OR “biomarkers” OR “clinical trials”). Inclusion criteria encompassed: (1) human studies; (2) randomized controlled trials, cohort studies, or systematic reviews; (3) articles with mechanistic or therapeutic focus. Exclusion criteria: (1) animal‐only studies; (2) case reports (<10 patients); (3) non‐English publications without validated translations.

This review integrates the latest advances in molecular pathogenesis, multiomics, and clinical trials to outline a roadmap for individualized care. Emphasizing the shift from a linear “multi‐hit” model to a dynamic self‐amplifying spiral, we link pathogenic mechanisms (mucosal dysregulation, Gd–IgA1 production, complement activation) to B‐cell, complement, and mucosal‐targeted therapies. We explore how novel biomarkers (e.g., Gd–IgA1, urinary CD163) enable dynamic risk stratification and “treat‐to‐target” (T2T) strategies. Concurrently, we critique controversies like Oxford MEST‐C score (Oxford classification (MEST‐C: mesangial hypercellularity [M], endocapillary hypercellularity [E], segmental sclerosis [S], tubular atrophy/interstitial fibrosis [T], crescent [C]) limitations and immunosuppression hazards, advocating evidence‐based approaches.

The review begins by underscoring the pressing need for more effective therapies through a detailed examination of IgAN's unfavorable long‐term prognosis, highlighting the persistent risk of progression to ESKD despite current standards of care. Building on this foundation, we systematically analyze the molecular mechanisms that drive disease progression, explicitly connecting these pathogenic insights to the therapeutic challenges faced in clinical practice. The subsequent sections critically appraise the latest evidence for supportive interventions, the clinical promise of novel targeted agents, and the potential of innovative modalities such as chimeric antigen receptor (CAT)‐T cell therapy, all within the context of evolving treatment paradigms. The review culminates in a pragmatic framework for initiating and monitoring therapy, emphasizing the integration of mechanistic and clinical data. By weaving together advances from bench to bedside, this work aims to empower clinicians to navigate IgAN's therapeutic transformation and advocates for the adoption of biomarker‐guided trial designs to resolve ongoing uncertainties and optimize patient outcomes.

## 2 Poor Prognosis and Global Burden of IgAN

IgAN imposes a substantial global health burden, with striking geographic disparities in prevalence and clinical outcomes. As the most common primary glomerulonephritis worldwide, IgAN accounts for 20–40% of biopsy‐proven glomerular diseases in Asia compared with 10–20% in Western populations [12]. Recent epidemiological studies estimate an annual incidence of 0.76 per 100,000 in Europe and 2.75 per 100,000 among Asian populations, with a global prevalence ranging from 2.53 per 10,000 in Europe to 10.5 per 100,000 in Australia [21, 22]. This geographic variation reflects genetic susceptibility, differential diagnostic practices, and environmental factors influencing mucosal immune dysregulation.

The economic impact of IgAN is profound, particularly in high‐risk populations. In the United States, patients with proteinuria ≥1 g/day incur nearly double the healthcare costs of those with lower proteinuria ($3732 vs. $1457 per patient per month) [23], driven by frequent hospitalizations and outpatient visits. Advanced chronic kidney disease (CKD) stages exacerbate this burden, with Stage 5 CKD costs exceeding $10,000 monthly. Mirroring this trend in China, the CK‐NET 2017–2018 report revealed that hospitalization costs for CKD patients averaged CNY 18,000 (∼$2600) per admission, with glomerular diseases like IgAN constituting 28.1% of all CKD hospitalizations [24]. Furthermore, ESKD patients requiring hemodialysis incurred annual expenses exceeding CNY 88,000 (∼$12,700), translating to over $1000 monthly—a catastrophic expenditure for 63% of households. Advanced CKD stages exacerbate this burden globally, with Stage 5 CKD costs exceeding $10,000 monthly in high‐income settings.

Recent long‐term follow‐up data from United Kingdom and Chinese cohorts have revealed a sobering prognosis for IgAN patients [18, 19], challenging the historical view of IgAN as a relatively benign condition. The UK National Registry of Rare Kidney Diseases study [18], analyzing 2299 adults and 140 children with IgAN, demonstrated that 50% of patients reached kidney failure or died within the study period, with a median kidney survival of 11.4 years. Most patients progressed to kidney failure within 10‐15 years across all age groups at diagnosis. Alarmingly, an annual estimated glomerular filtration rate (eGFR) decline of just 1 mL/min per 1.73 m^2^ per year would result in approximately 40% of adult patients younger than 50 years at diagnosis reaching kidney failure within their expected lifetime. These findings are corroborated by a large prospective cohort from China [19], which reported a median kidney survival time of 12.4 years, an annual event rate of ESKD of 41.1 per 1000 person‐years, and a 15‐year kidney survival rate of 40%. These studies suggest that up to 80% of patients may progress to kidney failure within 20 years [25, 26], underscoring the chronic and relentless nature of IgAN.

Notably, these poor outcomes persist despite advances in supportive care and the use of RAS inhibitors. Even patients with relatively low levels of proteinuria, traditionally considered with “low‐risk” (0.5–1 g/day), showed significant rates of kidney failure within 10 years [18, 26]. These findings highlight the urgent need for more effective, disease‐specific therapies and emphasize the importance of ongoing research into novel therapeutic approaches [27, 28, 29, 30, 31, 32, 33]. The recognition of IgAN as a disease with such poor long‐term outcomes underscores the critical need for earlier diagnosis, intervention, and better prognostic markers to identify high‐risk patients who might benefit from more aggressive treatment approaches early in the disease course.

## 3 Molecular Pathogenesis

### 3.1 The Multi‐Hit Hypothesis: Expanded and Updated

**FIGURE 1:**
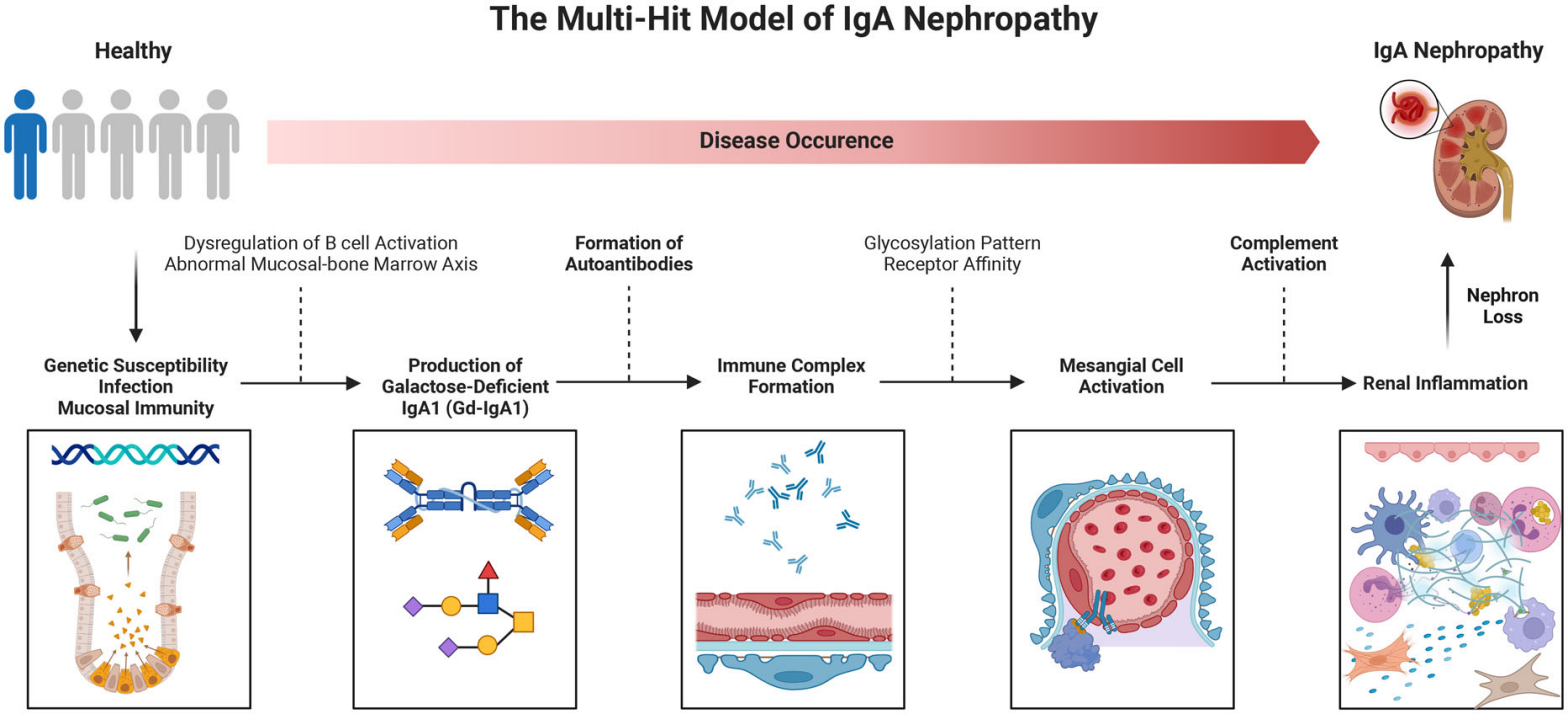
The multi‐hit pathogenesis model of IgA nephropathy. It illustrates the sequential pathogenic cascade in IgAN, known as the ”multi‐hit hypothesis.” The model delineates four interdependent stages driving disease progression: *Aberrant IgA1 production*: Dysregulated mucosal and systemic immunity triggers overproduction of Gd–IgA1, characterized by impaired O‐glycosylation in the hinge region. This creates a neoepitope recognized by the immune system. *Autoantibody generation*: Circulating autoantibodies (primarily IgG/IgA) target Gd–IgA1, forming pathogenic immune complexes (ICs). These ICs exhibit heightened nephritogenicity due to their biochemical properties. *Immune complex deposition*: ICs migrate to glomeruli and deposit in the mesangium via interactions with transferrin receptor 1 (CD71) and soluble CD89 (sCD89). This forms ternary complexes (IgA1–sCD89–CD71) that activate mTOR signaling, triggering mesangial cell proliferation. *Inflammatory injury*: Mesangial deposition initiates complement activation (primarily alternative and lectin pathways), cytokine release (IL‐6, TNF‐α), and recruitment of inflammatory cells. This cascade culminates in podocyte injury, matrix expansion, and progressive fibrosis. The model underscores Gd–IgA1 as the foundational abnormality, with each stage amplifying downstream pathological effects. Despite its explanatory value, this framework fails to fully capture IgAN's recurrent, self‐perpetuating nature—a limitation addressed by the “spiral hypothesis” in contemporary pathogenesis models.

The molecular pathogenesis of IgAN has traditionally been conceptualized through the “multi‐hit hypothesis,” which has significantly influenced therapeutic target discovery and drug development (Figure 1) [34, 35, 36, 37, 38]. This model outlines four sequential steps: (1) overproduction of Gd–IgA1 due to dysregulated mucosal and systemic immunity; (2) generation of autoantibodies against Gd–IgA1; (3) formation of circulating immune complexes (ICs); and (4) glomerular deposition of these ICs, leading to complement activation, mesangial cell proliferation, and inflammatory injury. Gd–IgA1, defined by aberrant O‐glycosylation in the hinge region, serves as a neoepitope that elicits autoantibody production and IC formation [39, 40, 41]. These immune complexes preferentially deposit in the mesangium via interactions involving IgA, transferrin receptor 1 (CD71), and soluble CD89 (sCD89), forming tripartite complexes (IgA1–sCD89–CD71) that activate mTOR signaling [42, 43, 44, 45, 46, 47, 48, 49, 50, 51, 52]. sCD89 promotes mesangial cell proliferation through PI3K/Akt/mTOR pathways, and glomerular sCD89 deposits correlate with histological markers of disease activity, especially in pediatric IgAN. Experimental models show that recombinant sCD89 injections recapitulate key IgAN features, such as mesangial hypercellularity and proteinuria, and in vitro studies confirm its direct role in triggering inflammatory cytokine release (e.g., IL‐6, TNF‐α) and complement activation [52, 53].

### 3.2 Integrating Recent Mechanistic Insights

While the multi‐hit hypothesis remains a cornerstone, recent multiomics and single‐cell studies have revealed a much more intricate interplay of immune and renal cells in IgAN pathogenesis. Expanded populations of mucosal‐associated invariant T cells and CD4+ T helper 17 (Th17) cells have been identified in IgAN patients, promoting B‐cell differentiation into IgA‐secreting plasmablasts via IL‐17 signaling pathways [54, 55, 56, 57, 58]. Mendelian randomization and immunophenotyping further implicate dysregulated B‐cell receptor signaling and impaired tolerance checkpoints in transitional B cells, with specific immunophenotypes (e.g., IgD+ CD24− B cells) correlating with disease risk due to altered energy metabolism and defective negative selection [59, 60, 61, 62]. Elevated BAFF/APRIL levels in IgAN sera drive autoreactive B‐cell survival, supporting these findings [63, 64, 65, 66, 67]. Beyond lymphocytes, renal parenchymal cells actively contribute to disease progression: mesangial cells exposed to Gd–IgA1–ICs upregulate Toll‐like receptor (TLR)4 and NLRP3 inflammasomes, activating MAPK/ERK and NF‐κB signaling, which leads to proinflammatory cytokine release (IL‐6, TNF‐α) [68, 69]. Glomerular immune complexes containing Gd–IgA1 stabilize alternative pathway (AP) complement activation through properdin‐mediated C3 convertase formation, amplifying mesangial injury via C5a receptor‐mediated inflammation and membrane attack complex (MAC)‐induced cell lysis; this also drives progressive tubulointerstitial fibrosis through C5a‐induced macrophage chemotaxis and endothelial cell activation [70, 71, 72, 73, 74, 75, 76, 77, 78, 79, 80, 81]. Complement activation in IgAN involves both alternative and lectin pathways, with CFH/CFHR genetic variants propelling AP dysregulation and C3a/C5a‐mediated inflammation perpetuating progression [82, 83, 84, 85, 86, 87, 88, 89, 90]. Urinary C3a/C5a levels correlate with histological severity and predict eGFR decline, while CFHR1/CFHR5 overexpression amplifies C3 convertase activity, creating a self‐sustaining injury loop. Therapeutic targeting of the complement axis is supported by clinical trials: iptacopan (a factor B inhibitor) reduces proteinuria by 38.5% and slows eGFR decline by 1.1 mL/min/year compared with placebo, while C5a receptor antagonists (such as avacopan) show similar efficacy in early‐phase studies [91]. Tubular epithelial cells exposed to sustained cytokine stimulation adopt a profibrotic phenotype via TGF‐β/Smad3 signaling, a process exacerbated by epigenetic modifications in fibrotic pathways, such as DNA hypermethylation of C1GALT1 [92, 93].

### 3.3 Sustained Pathogenic Cycles and Molecular Mimicry

The chronic progression of IgAN is perpetuated by a self‐reinforcing pathogenic cycle, in which persistent antigenic triggers and molecular mimicry play central roles. The “trigger hypothesis” posits that recurrent mucosal infections, such as streptococcal pharyngitis, provoke episodic surges in aberrant IgA production, which in turn drive disease flares. Recent studies have identified βII‐spectrin as a mesangial autoantigen, sharing structural homology with microbial antigens—particularly the SH3 domain of Streptococcus M proteins—thereby facilitating the formation of cross‐reactive IgA autoantibodies [94, 95]. This process is further exacerbated by gut dysbiosis: for example, overgrowth of *Akkermansia muciniphila* can degrade intestinal MUC1 via bacterial proteases, generating IgA1 neoepitopes that escape immune tolerance. In parallel, TLR9 activation in tonsillar B cells, as demonstrated in Japanese cohorts, induces APRIL overexpression, which sustains Gd–IgA1 synthesis [96, 97, 98, 99, 100, 101, 102].

### 3.4 Epigenetic and Posttranscriptional Regulation

Epigenetic dysregulation is increasingly recognized as a critical factor in IgAN pathogenesis. For instance, IL‐6–driven overexpression of GALNT14 impairs galactosylation of the IgA1 hinge region, while histone acetylation at TNFRSF13B (TACI) enhances BAFF/APRIL‐mediated plasma cell survival [103, 104, 105]. Furthermore, TRDMT1‐induced RNA 5‐methylcytosine (m5C) modification regulates IgA class switch recombination by modulating activation‐induced cytidine deaminase activity, and miR‐23b downregulation leads to increased pathogenic IgA synthesis [106, 107]. These epigenetic mechanisms create a vicious cycle; increased m5C modification exacerbates disease severity in mouse models, while pharmacological inhibition of m5C normalizes IgA levels and ameliorates renal injury.

### 3.5 Innate Immunity and the Renal Inflammatory Niche

Single‐cell multiomics analyses have revealed that these molecular alterations sustain a self‐perpetuating inflammatory niche within the kidney. M2c‐polarized macrophages secrete PDGFB to activate mesangial cells, while injured podocytes release HMGB1, which recruits CD163+ macrophages via TLR4, establishing a feedforward inflammatory loop [55, 57]. Clinically, this cycle is reflected in episodic disease flares following infection, with higher urinary sCD163 levels predicting steroid resistance, and glomerular CD206+ macrophage density correlating with treatment response [108, 109, 110]. These findings underscore the clinical utility of integrating urinary sCD163 and glomerular macrophage markers as biomarkers for individualized, niche‐targeted therapeutic strategies, enabling more precise risk stratification and tailored treatment selection.

### 3.6 Limitations of the Multi‐Hit Model

Each infectious trigger transiently elevates circulating pathogenic IgA, which, if not cleared, deposits in glomeruli, activates complement, and recruits inflammatory cells, resulting in a “hit‐and‐run” pattern of injury. Over time, repeated insults cumulatively reduce nephron reserve, accelerating progression to chronic kidney injury despite periods of clinical quiescence between flares. This paradigm mirrors the “trigger‐reload” dynamics observed in autoimmune diseases, where repeated antigenic exposures overwhelm regulatory mechanisms, transforming episodic attacks into relentless kidney destruction.

Despite these advances, significant knowledge gaps remain in our understanding of IgAN pathogenesis. The origin of Gd–IgA1 is still debated: while gut‐associated lymphoid tissue (GALT) is implicated via CCR9+ IgA+ plasmablasts homing to mucosal sites, bone marrow‐derived CD38hi CD27+ plasmablasts also contribute, as evidenced by clonal IgA1 expansions and shared somatic hypermutations between mucosal and systemic compartments in IgAN patients [57, 96, 111, 112, 113, 114, 115, 116, 117, 118, 119, 120, 121, 122, 123, 124, 125, 126, 127]. Single‐cell RNA sequencing has revealed CXCL12/CXCR4‐mediated crosstalk between these populations, suggesting a dual origin for pathogenic IgA1 [57, 128, 129, 130].

The role of innate immunity is also undercharacterized. Renal macrophages polarize toward M2c (CD163+ CD206+) and CD14+ CD16+ intermediate subsets, secreting TGF‐β1 and IL‐10 to drive fibrosis, while CD103+ dendritic cells promote Th17 differentiation via IL‐23/IL‐1β, collectively forming a profibrotic niche through PDGF‐B/PDGFRβ signaling and exacerbating mesangial matrix expansion [131, 132, 133, 134, 135, 136, 137, 138, 139, 140, 141, 142]. The renal microenvironment evolves into a self‐sustaining inflammatory circuit, with injured podocytes releasing HMGB1 that activates TLR4 on tubular epithelial cells, inducing chemokines such as CXCL1/IL‐8 to recruit S100A9+ neutrophils [143, 144, 145, 146, 147, 148]. These neutrophils release neutrophil extracellular traps (NETs) enriched in calprotectin (S100A8/A9) and IgA‐binding proteins, perpetuating immune complex deposition and complement activation via properdin stabilization [14, 149, 150]. Single‐cell multiomics identifies a feedforward loop where NET‐derived histones stimulate TLR9 in B cells, amplifying Gd–IgA1 production [57, 151, 152, 153, 154, 155, 156, 157, 158, 159, 160, 161, 162].

The multi‐hit pathogenesis framework, while foundational, remains incomplete. First, although elevated serum levels of Gd–IgA1 and Gd–IgA1‐specific IgG autoantibodies correlate with disease progression, standardized assays for these biomarkers lack universal clinical validation and their prognostic utility varies across populations [34, 163, 164, 165, 166, 167, 168, 169, 170]. Second, the complement activation paradox remains unresolved: although immune complexes containing IgG typically activate the classical pathway, IgAN predominantly involves alternative and lectin pathways. Recent evidence shows that lectin pathway components (e.g., mannose‐binding lectin [MBL], L‐ficolin) mediate glomerular injury in a subset of patients, with MBL–IgA1 complexes triggering C4d deposition independent of classical pathway activation [171, 172, 173]. Third, the model does not explain why elevated Gd–IgA1 levels in asymptomatic relatives often do not lead to IgAN, despite shared genetic predisposition [174]. Additional modifiers—such as T‐cell polarization patterns (Th17/Treg imbalance), innate immune cell activation, and tissue‐specific adaptation—may determine clinical progression [175].

Furthermore, the model's focus on circulatory factors underrepresents renal parenchymal responses—particularly mesangial cell epigenetic reprogramming and local complement activation—that actively shape disease phenotype, as demonstrated by single‐cell transcriptomics of IgAN biopsies [58, 176, 177, 178, 179]. The episodic recurrence of proteinuria despite initial immunosuppressive response, exemplified by several trials showing a 40% relapse rate within 3 years post steroid therapy, challenges the linear “hit accumulation” concept [180, 181, 182, 183]. These conceptual gaps underscore the necessity of expanding the paradigm to include tissue‐specific adaptation mechanisms and dynamic host–environment interactions across disease phases. Given these limitations, a new conceptual framework is required to capture the recurrent and dynamic nature of IgAN pathogenesis.

### 3.7 The Spiral Hypothesis: A Dynamic Model of IgAN Progression

**FIGURE 2:**
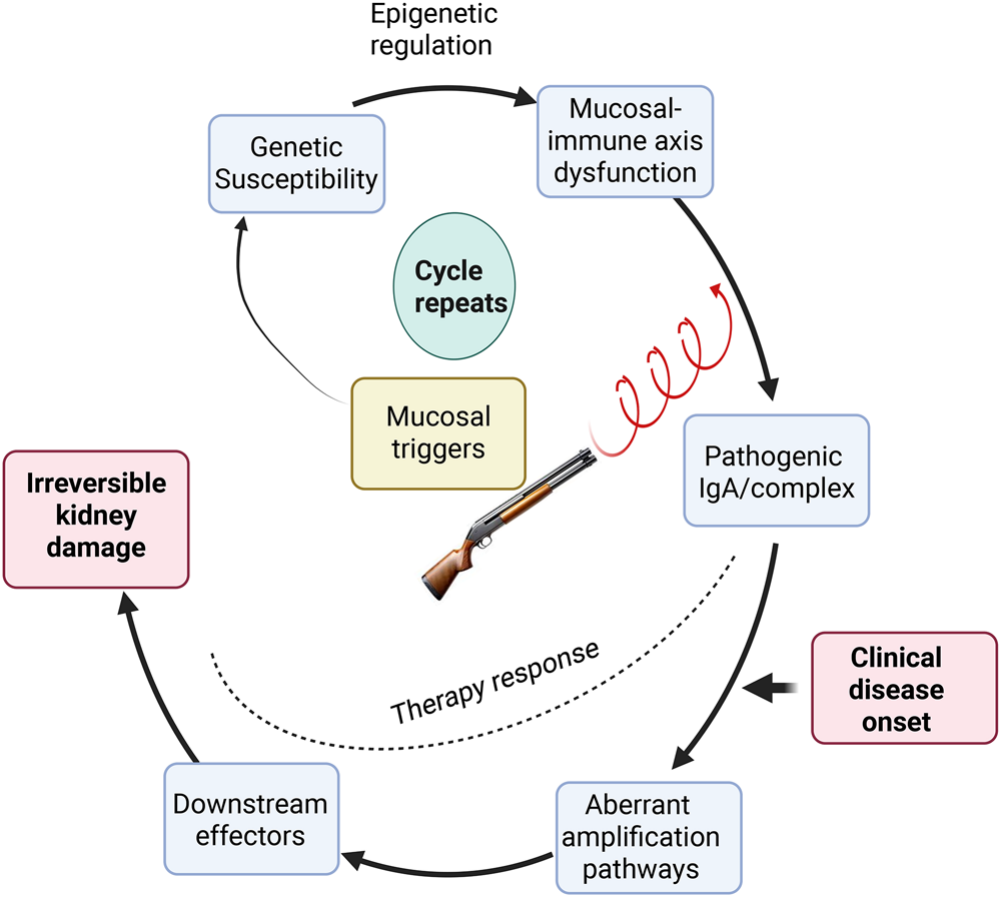
The spiral hypothesis of IgA nephropathy progression. This conceptual model illustrates the dynamic, self‐perpetuating nature of IgAN pathogenesis, moving beyond the traditional linear “multi‐hit” framework. The spiral represents recurrent cycles of mucosal immune dysregulation triggered by environmental factors (e.g., infections, dysbiosis), which initiate overproduction of pathogenic IgA and further pathogenic immune complexes that deposit in glomeruli, activating complement cascades (alternative/lectin pathways) and triggering mesangial inflammation. With each cycle, renal injury amplifies through interconnected feedback mechanisms: Tissue damage releases damage‐associated molecular patterns (DAMPs) such as HMGB1, recruiting profibrotic M2c macrophages that secrete PDGF‐B. Concurrently, injured podocytes sustain inflammation via TLR4 signaling, while epigenetic alterations (e.g., GALNT14 upregulation) perpetuate aberrant IgA1 glycosylation. Molecular mimicry—exemplified by cross‐reactivity between microbial antigens and renal autoantigens like βII‐spectrin—further fuels autoimmune responses. This self‐reinforcing circuit transforms transient insults into cumulative nephron loss, explaining disease progression during clinical remission. The model highlights the importance of targeting recurrent immune activation at mucosal sites, downstream inflammatory mediators, and fibrotic pathways to interrupt cyclical drivers—a strategy concordant with IgAN's relapsing‐remitting course.

Recent advances in single‐cell and multiomics studies, as well as clinical observations, suggest that the pathogenesis of IgA nephropathy is best conceptualized not as a simple linear cascade, but as a dynamic, self‐perpetuating spiral. We propose a “spiral hypothesis” (Figure 2), positing that IgAN progression involves repeated cycles of mucosal immune dysregulation, pathogenic IgA1 production, immune complex formation, and renal injury. Each cycle amplifies both systemic and local immune responses, leading to cumulative nephron loss and irreversible kidney damage over time. This model highlights the interplay between genetic susceptibility, epigenetic regulation, environmental triggers (such as infections and gut microbiota alterations), and renal tissue adaptation. The spiral is driven by recurrent mucosal triggers and sustained by tissue‐level inflammatory and fibrotic responses, resulting in a progressive decline in renal function even during periods of clinical remission. Importantly, this hypothesis underscores the need for therapeutic strategies that interrupt not only the initial pathogenic events but also the recurrent, amplifying cycles that characterize disease progression. By targeting both immune dysregulation and the chronic fibrotic milieu, the spiral hypothesis may provide a more comprehensive framework for understanding clinical heterogeneity and guiding personalized intervention in IgAN.

### 3.8 Integrative Summary

In summary, the molecular pathogenesis of IgAN is defined by a complex interplay between mucosal immune dysregulation, pathogenic IgA production, complement activation, and the establishment of a self‐sustaining renal inflammatory niche. While the multi‐hit hypothesis has provided a valuable roadmap for targeted therapy development, recent multiomics and single‐cell studies continue to reveal additional layers of complexity—including the roles of T cell subsets, innate immune mechanisms, epigenetic regulation, and the gut microbiome. The spiral hypothesis better encapsulates the chronic, relapsing, and self‐amplifying features of IgAN, calling for integrated therapeutic strategies that address both the triggers and the perpetuating cycles of immune‐mediated and fibrotic injury.

## 4 Current Therapy Dilemma

A paradigm shift has emerged in the management of IgAN, distinguishing between etiological therapies targeting disease mechanisms and general CKD treatments. This evolving approach has sparked considerable debate (Table 1), particularly regarding the optimal timing and appropriateness of initiating etiological treatments. The controversy reflects the complex and heterogeneous nature of IgAN, as well as the ongoing challenges in developing effective, targeted therapies for this common glomerular disease.

**TABLE 1:** Key considerations on early versus delayed intervention in IgAN.

Factor	Early intervention	Delayed intervention
Target population	High‐risk patients (e.g., proteinuria >1 g/day, active histology, elevated Gd–IgA1)	Low‐risk patients (e.g., proteinuria <0.5 g/day, MEST‐C score M0E0S0T0)
Rationale	Halts “multi‐hit” cascade before irreversible fibrosis; maximizes renal reserve	Avoids overtreatment in indolent cases; reduces drug exposure
Key benefits	30–40% reduction in ESKD risk; preserves eGFR slope	Lower healthcare costs; avoids immunosuppression‐related complications
Key risks	Potential for unnecessary treatment in self‐limiting cases; high drug costs	Missed window for disease modification; accelerated progression in occult high‐risk cases

Proponents of early intervention emphasize that addressing the underlying pathogenic mechanisms at an early stage could potentially alter the disease course and improve long‐term outcomes. This view is supported by accumulating evidence that early immunological events‐such as the production of Gd–IgA1 and the formation of immune complexes‐play a crucial role in disease initiation and progression. By intervening before significant renal damage occurs, early therapy may help prevent or delay the development of CKD and ESKD.

Conversely, some experts urge caution regarding the widespread use of etiological treatments, especially novel agents such as targeted‐release budesonide (Nefecon). Concerns have been raised about the limited long‐term efficacy data and the high costs associated with these therapies. The inclusion of budesonide in broad treatment recommendations has been particularly contentious, with critics arguing that the current evidence base is insufficient to support its widespread adoption as a first‐line therapy. These experts stress the importance of optimizing supportive care‐such as blood pressure control, proteinuria reduction, and cardiovascular risk management‐before considering more aggressive immunomodulatory treatments.

The introduction of sparsentan (US FDA‐approved 2023) [184] and iptacopan (accelerated approval 2024) [185] has reshaped the therapeutic landscape. Sparsentan, a dual ET–angiotensin receptor antagonist, reduces proteinuria by 42.8 versus 4.4% with irbesartan at 110 weeks while preserving eGFR (−2.9 vs. −3.9 mL/min/year). Iptacopan [186], an alternative complement pathway inhibitor, achieves 35% proteinuria reduction and eGFR stabilization in C3d‐positive patients. These agents, alongside targeted‐release budesonide, intensify challenges in patient selection and treatment timing.

Given the heterogeneous clinical presentations and variable progression rates of IgAN, determining which patients are most likely to benefit from specific therapies, and at what point in their disease course, remains a significant challenge [187, 188]. The optimal use of these agents—whether as monotherapy or in combination with existing or other novel treatments—has yet to be fully established.

The complex pathogenesis of IgAN, involving multiple “hits” to the immune system and kidneys, suggests that combination therapies targeting different pathways may offer synergistic benefits. For example, combining supportive care with targeted therapies could potentially enhance efficacy. However, such approaches must be balanced against the risk of increased side effects and higher treatment costs.

The controversy between systemic corticosteroids and TRF‐budesonide centers on efficacy‐safety trade‐offs (Table 2). While the TESTING trial demonstrated significant eGFR preservation with reduced‐dose methylprednisolone (−2.7 mL/min/year), it reported 16.5% serious infections and 2.1% mortality [182, 183]. In contrast, NefIgArd showed comparable proteinuria reduction but superior long‐term eGFR preservation (−2.1 vs. −7.5 mL/min/year in placebo) without steroid‐specific complications [186, 189, 190]. This divergence underscores the value of tissue‐targeted approaches.

**TABLE 2:** Efficacy and safety comparison between TRF‐budesonide and systemic corticosteroids.

Parameter	Systemic corticosteroids (TESTING trial—reduced dose)	TRF‐budesonide (NefIgArd trial)	Clinical implications
Dosing protocol	Oral methylprednisolone (0.4 mg/kg/day, max 32 mg/day), tapered over 6–9 months	16 mg/day for 9 months	Targeted delivery minimizes systemic exposure
Proteinuria reduction	51% at 12 months	31% at 9 months; 50% at 12 months (posttreatment)	Comparable early efficacy; TRF shows sustained posttreatment benefit
eGFR preservation	−2.7 mL/min/year (reduced‐dose arm)	−2.1 vs. −7.5 mL/min/year (placebo) at 24 months	Superior long‐term renal protection with TRF
Serious adverse events	Reduced‐dose arm: lower infection risk vs. high‐dose (16.5% serious infections in high‐dose arm)	9.2% (vs. 5.8% placebo)	Threefold lower infection risk with TRF vs. systemic steroids
Steroid‐specific toxicity	New‐onset diabetes: 8.7% (high‐dose); no data for reduced‐dose	Prediabetes progression: 1.8%; no diabetes/fractures	Favorable metabolic/bone safety profile for TRF
Discontinuation rate	7.7% (reduced‐dose) vs. 15.3% (high‐dose)	9% (vs. 2% placebo)	Improved tolerability of reduced‐dose steroids vs. historical high‐dose regimens

Not all patients with IgAN necessarily require etiological treatment. Real‐world evidence and recent guideline updates indicate that patients with mild histological changes or stable, low‐level proteinuria may achieve long‐term stability with optimized supportive care alone [191, 192]. For instance, the TESTING trial run‐in phase revealed that 81.8% of patients maintaining proteinuria <1 g/day on RASi avoided progression without immunosuppression [193]. Although supportive therapies reduce proteinuria by 30–50% and slow CKD progression, longitudinal cohort studies indicate that 30–40% of patients still progress to ESKD within 20 years despite optimal management [194]. This underscores the need for additive targeted therapies in high‐risk populations, particularly those with active immunological markers (elevated Gd–IgA1, urinary CD163) or aggressive histology (crescents, C4d deposition) [195].

The advent of new therapies also raises questions about the sequencing and potential combination of treatments. While combination regimens may offer additive benefits, they also necessitate careful monitoring for overlapping toxicities and cost‐effectiveness. Emerging data suggest synergistic effects when combining supportive and targeted therapies. For example, the SPARTACUS trial found that adding sparsentan to sodium‐glucose cotransporter‐2 inhibitors (SGLT2is) yielded additive proteinuria reduction compared with SGLT2i alone [196]. Conversely, the NefIgArd open‐label extension showed that combining Nefecon with iptacopan‐targeting both Gd–IgA1 production and complement‐mediated injury‐reduced proteinuria more efficiently while stabilizing eGFR [197, 198]. However, such regimens require careful monitoring for overlapping toxicities (e.g., infections with complement inhibitors, fluid retention with ET antagonists).

Furthermore, the personalization of treatment strategies is likely to become increasingly important as more therapeutic options become available. Factors such as genetic predisposition, biomarker profiles, and histological findings may play crucial roles in guiding individualized treatment decisions. Elevated serum APRIL levels may predict a response to B‐cell‐targeted therapies like sibeprenlimab, while urinary C3a/C5a ratios correlate with complement inhibitor efficacy. Ongoing phase 3 trials of atrasentan (selective ET‐A antagonist) and zigakibart (APRIL inhibitor) will further clarify the roles of mechanistically distinct agents [199, 200, 201, 202, 203, 204]. Real‐time predictive models that incorporate these factors, alongside clinical parameters, could help tailor therapy to individual patients, maximizing efficacy while minimizing unnecessary exposure to potential side effects.

Ultimately, the goal is to balance immunological control with preservation of renal reserve—a task complicated by IgAN's heterogenous pathogenesis. This heterogeneity necessitates divergent approaches: for chronic, slowly progressive disease, experts advocate step‐up therapy with minimal sequential agents to avoid overtreatment; conversely, in rapidly progressive subgroups, early intensive immunosuppression is critical to counter irreversible glomerulosclerosis. While early intervention in high‐risk patients appears justified, overtreatment of indolent cases remains a concern [20]. Looking ahead, well‐designed clinical trials comparing treatment strategies—including de‐escalated regimens for low‐risk patients and combination therapies for high‐risk subtypes—will be essential. These studies should address not only short‐term outcomes like proteinuria reduction but also long‐term renal survival and quality of life. Additionally, real‐world evidence tracking risk‐stratified implementation of new therapies will be crucial for refining treatment algorithms.

In summary, while IgAN remains a challenging disease with significant unmet needs, the rapidly evolving therapeutic landscape offers new hope for patients and clinicians. The coming years will be critical in determining how best to integrate these novel therapies into clinical practice, with the ultimate goal of improving renal outcomes and quality of life for patients with this common but potentially devastating kidney disease. As research progresses, the prospect of transforming IgAN from a disease with an uncertain prognosis to a manageable chronic condition with preserved kidney function may become a reality for many patients.

## 5 Supporting Therapies

Supportive therapy remains the cornerstone of IgAN management, forming the essential foundation upon which all other interventions are built [205, 206, 207]. Despite significant advances in disease‐specific therapies, optimized supportive care—including lifestyle modification, blood pressure control, and RASi—continues to be the first‐line approach for all patients, irrespective of risk stratification [15, 17, 20, 207, 208, 209, 210]. This strategy aims not only to slow the progression of CKD but also to reduce cardiovascular morbidity, which constitutes a major cause of mortality in CKD population.

Contemporary treatment algorithms prioritize a stepwise approach: initial optimization of supportive care for 3–6 months, followed by escalation to targeted therapies in nonresponders. As outlined by Praga et al. [211], this therapeutic hierarchy initiates supportive care with RAS inhibition ± SGLT2i for proteinuria >0.5 g/day, reserving ERAs or immunosuppression for patients with persistent proteinuria >1 g/day despite optimized supportive therapy, histological risk (MEST‐C ≥2), and/or progressive eGFR decline.

The primary therapeutic objectives of supportive care are to achieve rigorous blood pressure control (generally targeting <130/80 mmHg, and <120 mmHg for select high‐risk patients), minimize proteinuria (ideally to <0.5 g/day), and address modifiable lifestyle factors such as obesity, smoking, and dietary sodium intake [195, 212]. The efficacy of RAS inhibitors (ACEi/ARB) in reducing proteinuria and preserving renal function is well established [213, 214, 215, 216], with multiple randomized controlled trials and meta‐analyses showing that maximally tolerated doses confer significant renal protection compared with other antihypertensive agents or placebo. Recent evidence also supports the addition of SGLT2is for patients with persistent proteinuria or declining eGFR despite optimal RAS blockade [217, 218, 219, 220], with subgroup analyses from DAPA‐CKD and EMPA‐KIDNEY demonstrating substantial reductions in kidney disease progression and ESKD risk in IgAN cohorts.

Practical integration of SGLT2i faces real‐world challenges: cost barriers in low‐resource settings, eGFR eligibility thresholds (typically >20 mL/min), and management of genital infections. Nonetheless, their cardiorenal benefits position them as second‐line agents after RASi, particularly for patients with eGFR decline >2 mL/min/year despite optimized supportive care.

Contemporary supportive care extends beyond traditional agents to incorporate newer pharmacological modalities with pleiotropic effects. ERAs like sparsentan and mineralocorticoid receptor antagonists (MRAs) exhibit dual antiproteinuric and antifibrotic properties through TGF‐β pathway modulation [184, 200, 201, 221, 222, 223, 224, 225, 226, 227, 228, 229, 230, 231, 232, 233, 234, 235]. Though mechanistically novel, these agents primarily target downstream pathological processes rather than underlying disease etiology. Their integration into treatment regimens exemplifies the evolution of supportive strategies, complementing rather than replacing foundational RAS inhibition. Sparsentan (US FDA‐approved 2023) now integrates into algorithms for persistent proteinuria without hematuria, while MRAs like finerenone require cautious potassium monitoring, limiting use in advanced CKD. Real‐world implementation is challenged by drug–drug interactions (e.g., NSAIDs) and the absence of head‐to‐head trials against SGLT2i.

The immunomodulatory potential of supportive therapies also warrants emphasis. RAS inhibitors mitigate NF‐κB‐driven inflammation and TGF‐β‐mediated fibrosis, while SGLT2i suppress NLRP3 inflammasome activation and macrophage recruitment [236, 237, 238, 239, 240, 241]. These ancillary properties may explain the 30–40% sustained remission rate observed with optimized supportive care alone. The STOP‐IgAN trial paradigm further validates this approach [205], demonstrating that 6 months of intensive supportive management enables 40% of initially eligible patients to avoid immunosuppression without compromising outcomes.

For disease‐modifying agents (TRF‐budesonide, BAFF/APRIL inhibitors), supportive care remains the mandatory backbone. Nefecon is prioritized for elderly/comorbid patients intolerant to steroids, while complement inhibitors (e.g., iptacopan) require C3d/Gd–IgA1 biomarkers for patient selection—a significant barrier in regions lacking specialized assays. Cost remains prohibitive: annual pricing exceeds $70,000 for novel agents versus $500–$1200 for RASi/SGLT2i combinations.

It is reasonable to endorse a 1–3 month observation period with optimized supportive therapy before considering immunomodulation, except in cases with high‐risk features: active histological lesions (crescents >25%, fibrinoid necrosis), nephrotic‐range proteinuria (>3.5 g/day), or rapid eGFR decline (>5 mL/min/1.73 m^2^/year) [207]. This strategic delay allows assessment of treatment responsiveness while minimizing early exposure to immunosuppressive risks.

Nevertheless, even with best‐practice supportive management, a considerable residual risk of CKD progression persists, particularly among patients with persistent proteinuria or high‐risk pathological features. This underscores the need for adjunctive disease‐modifying therapies but also reinforces the principle that supportive care should be maintained as the backbone of all treatment regimens. The integration of novel agents—such as targeted‐release budesonide [186, 190] and complement inhibitors [185]—into the therapeutic landscape does not diminish the foundational role of supportive care; rather, these agents are layered atop optimized supportive measures to maximize renal preservation.

Critical implementation gaps persist: (1) biomarker accessibility limits precision deployment of APRIL inhibitors (e.g., sibeprenlimab requires serum APRIL levels); (2) long‐term safety data for combination regimens (e.g., SGLT2i + ERA) remains incomplete; (3) rural/underserved regions lack infrastructure for rigorous proteinuria monitoring. These disparities necessitate context‐adapted protocols: urban centers may adopt biomarker‐guided escalation, while community clinics prioritize RASi/SGLT2i combinations with quarterly monitoring.

Ultimately, the sustained success of any intervention in IgAN hinges on the continued optimization of supportive therapy as the platform for all subsequent, individualized treatment strategies. As research advances and disease‐modifying therapies become increasingly available, the transition from optimized supportive care to etiology‐targeted interventions should be guided by persistent proteinuria, progressive decline in kidney function, or the presence of high‐risk pathological features. In this way, supportive care not only maximizes the efficacy of new therapies but also ensures that patients receive the most appropriate and effective management at every stage of their disease.

## 6 Emerging Targeted Therapies

### 6.1 Targeted‐Release Formulation of Budesonide (TRF‐Budesonide)

**FIGURE 3:**
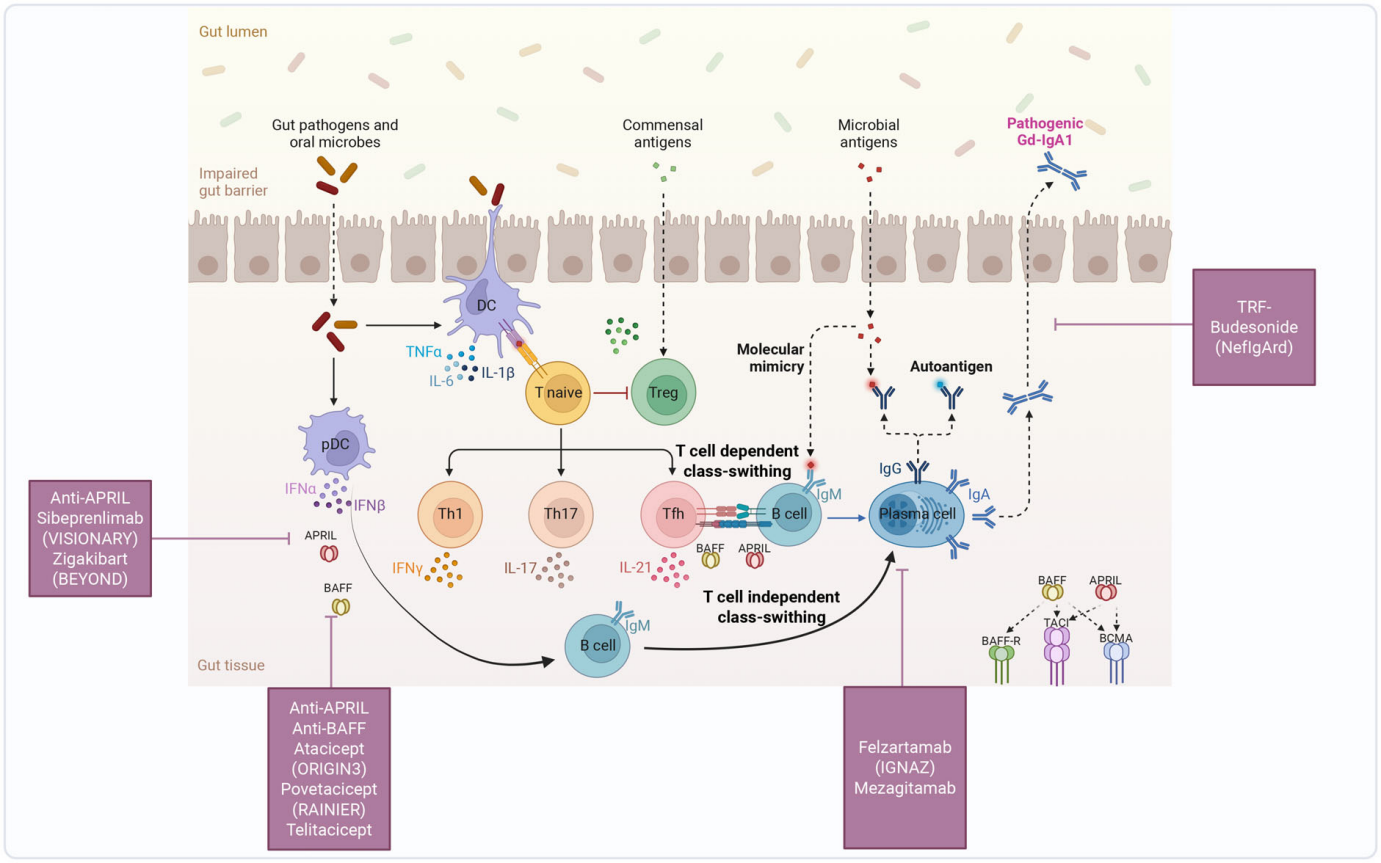
Therapeutic targeting of mucosal and B‐cell pathways in IgA nephropathy. This figure illustrates therapeutic strategies targeting the mucosal origins of pathogenic IgA production and systemic B‐cell dysregulation in IgAN. The upper section depicts mucosal immunomodulation via targeted‐release budesonide (Nefecon), which acts locally in the ileal Peyer's patches to suppress B‐cell activation and differentiation into IgA‐secreting plasma cells. By inhibiting NF‐κB signaling, Nefecon reduces systemic levels of galactose‐deficient IgA1 (Gd–IgA1) while minimizing systemic glucocorticoid exposure. The lower section details systemic B‐cell interventions: APRIL/BAFF inhibition (e.g., sibeprenlimab, telitacicept) blocks cytokine‐mediated B‐cell maturation and autoreactive plasma cell survival. CD38‐targeted depletion (e.g., felzartamab) eliminates antibody‐secreting plasma cells directly. These approaches synergistically disrupt pathogenic IgA production at distinct nodes: mucosal Gd–IgA1 overproduction and systemic autoantibody generation. The integrated strategy addresses IgAN's dual pathogenesis—mucosal immune dysregulation and B‐cell autoreactivity—particularly in patients with elevated Gd–IgA1 or active renal inflammation.

Targeted‐release formulation of budesonide (TRF‐budesonide, Nefecon) represents a significant advancement in IgAN treatment [186, 189, 190], addressing its underlying pathogenesis through localized delivery to GALT in the distal ileum—where >70% of mucosal IgA‐producing cells reside. This innovative approach targets the “mucosal origin” hypothesis of IgAN, where dysregulated IgA production occurs in Peyer's patches. Upon ingestion, Nefecon's pH‐dependent enteric coating dissolves in the distal ileum, releasing budesonide directly to GALT. This locally suppresses B‐cell activation, Gd–IgA1 production, and subsequent immune complex formation, while minimizing systemic exposure (Figure 3). By interrupting the disease process at its source, TRF‐budesonide offers a mechanistically precise alternative to broad immunosuppression.

The phase 3 NefIgArd trial demonstrated Nefecon's efficacy [186], showing a 31% reduction in proteinuria compared with placebo after a 9‐month treatment course. Additionally, the study revealed a statistically significant difference in eGFR decline between the Nefecon and placebo groups over a 2‐year period. Notably, the 2‐year eGFR benefit (−2.1 vs. −7.5 mL/min/year in placebo) persisted 9 months posttreatment cessation, suggesting disease‐modifying effects beyond the treatment window. The safety profile of Nefecon was further elucidated during a 15‐month observational follow‐up, where the incidence of treatment‐emergent adverse events (TEAEs) was similar between Nefecon and placebo groups, suggesting that adverse effects are largely confined to the active treatment period.

Common TEAEs associated with Nefecon included peripheral edema (17 vs. 4% placebo), hypertension (12 vs. 3%), muscle spasms (12 vs. 4%), acne (11 vs. 1%), and headache (10 vs. 8%). Of note, four prediabetic participants progressed to diabetes during the 9‐month treatment period, highlighting the importance of glucose monitoring in at‐risk patients. Critically, no instances of adrenal insufficiency, cataracts, or osteoporotic fractures—frequent complications of systemic steroids—were observed. The discontinuation rate due to TEAEs was 9% in the Nefecon group compared with 2% in the placebo group. While this higher rate is notable, it should be contextualized within Nefecon's overall risk–benefit profile, considering its demonstrated efficacy in preserving renal function and reducing proteinuria in IgAN patients.

Cost‐effectiveness analyses reveal nuanced economic implications. At an annual cost of ∼$70,000, Nefecon achieves an incremental cost‐effectiveness ratio (ICER) of $98,000/QALY versus supportive care in high‐risk patients—below the $150,000/QALY US willingness‐to‐pay threshold. However, this exceeds ICERs for SGLT2 inhibitors ($45,000/QALY) and RASi ($12,000/QALY). Budget impact models indicate potential savings by reducing ESKD incidence: treating 100 patients with Nefecon may prevent eight cases of dialysis ($2.4  M savings over 5 years), offsetting 40% of drug costs.

Several critical questions remain regarding the optimal use of TRF‐budesonide in IgAN. A key area for further investigation is the direct comparison between TRF‐budesonide and low‐dose systemic corticosteroids to clarify their relative efficacy and safety [182, 183]. While the TESTING trial demonstrated greater proteinuria reduction with systemic steroids, this came at the cost of threefold higher infection rates and significant metabolic toxicity. The NefIgArd extension data suggest TRF's superior long‐term eGFR preservation despite lesser acute proteinuria reduction, highlighting a fundamental efficacy‐safety trade‐off.

While the initial 9‐month treatment course with Nefecon has shown promising results, including a 31% reduction in proteinuria, the durability of its effects and the need for repeated or maintenance therapy remain uncertain. The NefIgArd open‐label extension showed proteinuria rebounded to 82% of baseline 15 months posttreatment, suggesting potential need for retreatment cycles in nonresponders. Long‐term safety data are also essential, particularly concerning extended or repeated treatment courses, even though its targeted delivery system is designed to minimize systemic side effects.

The potential for combining TRF‐budesonide with other immunosuppressive therapies also warrants exploration. Given the complex pathogenesis of IgAN, a multitargeted approach may enhance outcomes by addressing different aspects of the disease. For example, combining TRF‐budesonide with APRIL inhibitors (e.g., sibeprenlimab) or complement blockers (iptacopan) may synergistically target upstream Gd–IgA1 production and downstream inflammatory injury. However, careful evaluation of these combinations is necessary to ensure they do not compromise the favorable safety profile of TRF‐budesonide, particularly regarding risks like infections or metabolic disturbances.

Another priority is identifying patient subgroups most likely to benefit from TRF‐budesonide, either as monotherapy or in combination regimens. Biomarkers such as elevated serum Gd–IgA1, urinary CD163, and mucosal‐homing CCR9+ plasmablasts may help refine patient selection and monitor treatment response. Personalized approaches could optimize outcomes by targeting therapy to those at highest risk of progression while avoiding unnecessary treatment in lower‐risk patients.

### 6.2 B‐Cell Targeted Therapies

Recent advances in understanding the molecular pathogenesis of IgAN have catalyzed the development of B cell‐targeted therapies [121, 166, 242, 243], which can be broadly categorized into two strategic approaches (Figure 3): direct B‐cell depletion and modulation of B‐cell activation pathways. These interventions aim to disrupt the production of Gd–IgA1, a pivotal pathogenic factor in IgAN. Below, we synthesize current evidence and emerging directions, incorporating critical insights from recent literature.

#### 6.2.1 Direct B‐Cell Depletion Strategies

This approach seeks to reduce the populations of B cells and plasma cells responsible for pathogenic Gd–IgA1 production:

##### 6.2.1.1 Tonsillectomy

Historically considered for its potential to eliminate mucosa‐associated lymphoid tissue (MALT) sources of aberrant IgA, tonsillectomy remains controversial. While Japanese cohort studies initially suggested reduced proteinuria and improved renal outcomes [244, 245, 246], subsequent meta‐analyses reveal inconsistent benefits across diverse populations. Notably, a 2024 systematic review highlighted significant heterogeneity in patient selection and outcome measures, underscoring the lack of consensus on its therapeutic role. Given these limitations and the irreversible nature of the intervention, tonsillectomy cannot be routinely recommended outside select research contexts.

##### 6.2.1.2 GALT Targeting

The development of targeted‐release budesonide (NEFECON) represents a paradigm shift in modulating mucosal immunity. By delivering budesonide to the distal ileum—a hub for Peyer's patches and IgA class‐switching—this agent reduces local B‐cell activation without systemic immunosuppression. The phase 3 NefIgArd trial demonstrated sustained proteinuria reduction (48% at 12 months) and eGFR preservation, positioning it as the first US FDA‐approved therapy for IgAN. This spatially precise approach exemplifies how anatomic targeting can optimize efficacy while minimizing off‐tissue effects.

##### 6.2.1.3 CD20 Monoclonal Antibodies

The use of CD20 monoclonal antibodies, such as rituximab, in the treatment of progressive IgAN has not shown consistent benefit and is not currently recommended [247, 248]. The failure of CD20 monoclonal antibodies in IgAN may be attributed to several factors, including the persistence of long‐lived plasma cells that continue to produce pathogenic IgA1 even after B‐cell depletion, and the potential role of other immune cells in disease progression. This experience emphasizes the need for a more nuanced understanding of the immune dysregulation in IgAN and the importance of developing targeted therapies that address the specific pathogenic mechanisms of the disease.

##### 6.2.1.4 CD38/CD40 Pathway Inhibition

The use of monoclonal antibodies targeting CD38 or CD40, molecules expressed on B cells and plasma cells, represents a more systemic approach to B cell depletion [249, 250]. CD38 is highly expressed on plasma cells, which are responsible for the bulk of antibody production, including pathogenic Gd–IgA1 in IgAN. Monoclonal antibodies against CD38, such as daratumumab, felzartamab, have shown efficacy in multiple myeloma and are being investigated for their potential in IgAN. Early‐phase IgAN trials demonstrate proteinuria reductions (40–60% with felzartamab) but reveal significant toxicity—including cytopenias, opportunistic infections, and cardiotoxicity—offsetting potential benefits while biopsy efficacy awaits confirmation. The plasma cell depletion rationale targeting Gd–IgA1 producers remains biologically plausible despite safety challenges. Similarly, CD40 is a crucial molecule for B cell activation and survival, and blocking CD40–CD40L interactions with monoclonal antibodies could potentially disrupt the production of pathogenic antibodies. However, these approaches are still in early stages of investigation for IgAN (NCT05065970), and their efficacy and safety profiles need to be carefully evaluated, particularly given the broad effects these therapies may have on the immune system.

#### 6.2.2 Modulation of B‐Cell Activation

The second major strategy in targeting B cells in IgAN focuses on modulating B cell activation and the pathways involved in the abnormal production of IgA [251, 252, 253]. This approach aims to fine‐tune the immune response rather than broadly depleting B cells, potentially offering a more nuanced intervention with fewer systemic effects.

##### 6.2.2.1 Glucocorticoids

Glucocorticoids have long been used in the treatment of IgAN, and their effects on B cell function are well documented. They act through multiple mechanisms, including suppression of B cell proliferation, induction of apoptosis in activated B cells, and modulation of cytokine production. While systemic glucocorticoids have shown efficacy in reducing proteinuria and slowing disease progression in some patients with IgAN, their use is limited by significant side effects associated with long‐term systemic exposure. The development of targeted‐release formulations, as mentioned earlier, aims to harness the beneficial effects of glucocorticoids on mucosal B cells while minimizing systemic exposure.

##### 6.2.2.2 TLR Modulation

The modulation of TLR signaling represents a novel approach to regulating B cell activation in IgAN. TLRs play a crucial role in innate immunity and can influence B cell activation and antibody production [112, 254, 255, 256, 257, 258]. In IgAN, there is evidence of aberrant TLR signaling contributing to the production of Gd–IgA1 and the overall inflammatory milieu. TLR antagonists are being investigated as potential therapies to dampen this inappropriate immune activation. By targeting specific TLRs implicated in IgAN pathogenesis, such as TLR9, researchers hope to develop more targeted therapies that can modulate B cell function without broadly suppressing the immune system. Hydroxychloroquine (HCQ) has emerged as a promising treatment for IgAN, with its potential mechanism of action of interfering with TLR signaling by directly binding to nucleic acids and inhibiting endosomal TLR activation, particularly TLR9. A randomized controlled trial showed that HCQ, when added to optimized RAAS inhibition, significantly reduced proteinuria by 48.4% over 6 months compared with placebo. Moreover, HCQ exhibited a favorable safety profile with no serious adverse events reported during short‐term use [259, 260, 261]. However, the long‐term renoprotective efficacy and safety of HCQ in IgAN require further investigation. Future research should focus on larger, ethnically diverse randomized controlled trials with extended follow‐up periods to confirm HCQ's long‐term benefits and potential role as an alternative or adjunct to conventional immunosuppressive therapies in IgAN management.

##### 6.2.2.3 Mycophenolate Mofetil

Mycophenolate Mofetil (MMF) represents an immunomodulatory approach that regulates B‐cell activation and may contribute to reducing pathogenic IgA production in IgAN, though direct evidence for IgA suppression remains limited. By selectively inhibiting inosine monophosphate dehydrogenase, MMF impedes de novo purine synthesis, reducing B‐lymphocyte proliferation and plasma cell differentiation. While the precise impact on Gd–IgA1 antibodies is inferred rather than directly proven, clinical studies demonstrate MMF's efficacy in reducing proteinuria and stabilizing renal function in progressive IgAN [262, 263, 264, 265, 266, 267, 268, 269, 270, 271, 272, 273, 274], particularly in patients with active inflammation. The MAIN trial demonstrated significant advantages [269]: MMF combined with supportive care reduced the composite outcome risk by 77% (adjusted hazard ratio 0.23) and slowed eGFR decline (1.2 vs. 3.8 mL/min/1.73 m^2^/year) versus supportive care alone in progressive IgAN. This positions MMF as a viable strategy for modulating aberrant B‐cell responses without broad immunosuppression. Key limitations include rapid loss of renal protection after discontinuation and unresolved questions regarding long‐term safety and generalizability beyond Asian populations. MMF's favorable risk–benefit profile during treatment offers a validated alternative to broad immunosuppression, though its specific mechanistic impact on pathogenic IgA requires further validation.

##### 6.2.2.4 Proteasome Inhibitors

Proteasome inhibitors represent another innovative approach to modulating B cell function in IgAN. These drugs, such as bortezomib, target the proteasome, a cellular machinery crucial for protein degradation and cell signaling. In the context of B cells and plasma cells, proteasome inhibition can lead to the accumulation of misfolded proteins, triggering the unfolded protein response and ultimately leading to cell death. This approach has shown efficacy in multiple myeloma and is being explored in IgAN due to its potential to selectively target long‐lived plasma cells that may be resistant to other forms of B cell‐directed therapy [275, 276, 277]. Early studies in IgAN have shown promising results in reducing proteinuria [278, 279, 280, 281], but cytopenias and neuropathy limit long‐term use. Larger clinical trials are needed to establish the efficacy and safety of this approach in the long‐term management of the disease. Next‐generation inhibitors (e.g., carfilzomib) with improved safety profiles are being explored.

##### 6.2.2.5 APRIL/BAFF Pathway Inhibition

Targeting the APRIL (a proliferation‐inducing ligand) and BAFF pathways has emerged as a promising strategy in modulating B cell activation in IgAN [282, 283, 284]. These cytokines play critical roles in B cell survival, maturation, and antibody production. Elevated levels of APRIL and BAFF have been observed in patients with IgAN, suggesting their involvement in disease pathogenesis. Monoclonal antibodies targeting these cytokines or their receptors are under investigation as potential therapies for IgAN. By interrupting these signaling pathways, the goal is to reduce the survival and activation of autoreactive B cells producing pathogenic Gd–IgA1.

Among the investigational agents, sibeprenlimab, an APRIL‐specific inhibitor, demonstrated promising interim results in the phase 3 APPLAUSE‐IgAN trial (NCT05248646) [282, 283, 284], reporting a 62% reduction in proteinuria at 12 months. Telitacicept, a novel dual inhibitor of BAFF and APRIL, achieved a 49% proteinuria reduction at a 240 mg dosage over 24 weeks in phase 2 studies [285, 286]. Similarly, povetacicept, another dual BAFF/APRIL antagonist, yielded over 60% proteinuria reduction at 36 weeks in phase 1/2 trials [287], alongside associated remission and stable renal function. These agents collectively reduce Gd–IgA1 levels and exhibit safety profiles comparable to placebo, positioning them as potential first‐line disease‐modifying therapies. The compelling efficacy and tolerability have accelerated further development, with multiple BAFF/APRIL inhibitors advancing into phase 3 trials. If approved, these therapies could redefine standard care for IgAN (Tables 3 and 4), offering substantial clinical benefits for patients with this progressive kidney disease.

**TABLE 3:** Key B‐cell targeted therapies in IgA nephropathy.

Therapy category	Mechanism of action	Current evidence	Clinical stage
CD20 inhibitors	Depletes B cells (not plasma cells)	No significant proteinuria/eGFR benefit; not recommended (rituximab trials)	Phase 2 (failed)
CD38 inhibitors	Depletes plasma cells	Felzartamab: 40–60% proteinuria reduction in early trials; mezagitamab under study	Phase 2
APRIL inhibitors	Neutralizes APRIL, reducing plasma cell survival	Sibeprenlimab: 62% proteinuria reduction (phase 3 interim); reduces Gd–IgA1	Phase 3 (APPLAUSE‐IgAN)
BAFF/APRIL dual inhibitors	Blocks both BAFF and APRIL signaling	Telitacicept: 49% proteinuria reduction (phase 2); povetacicept: >60% reduction	Phase 3 (OPTIMIST)
Proteasome inhibitors	Induces plasma cell apoptosis	Bortezomib: 54% proteinuria reduction in small cohorts; limited by toxicity	Phase 2

**TABLE 4:** Overview of the current landscape of some treatments for IgAN, showcasing their effectiveness in reducing proteinuria and their impact on eGFR decline.

Drug name	Mechanism of action	Drug dose	Proteinuria reduction (%)	Annual eGFR decline rate under regimen (mL/min/year)	Annual eGFR decline rate in placebo (mL/min/year)
Methylprednisolone	Systemic corticosteroids act through multiple pathways: suppression of B cell proliferation, induction of apoptosis in activated B cells, modulation of cytokine production.	0.4 mg/kg/d (Reduced‐dose)	∼35	−2.5	−5.0
TRF‐budesonide	Acts locally at Peyer's patches in the distal ileum to reduce immune response and formation of autoantibodies	16 mg/d	30.1	−3.1	−6.0
Sibeprenlimab	Sibeprenlimab is a monoclonal antibody that blocks APRIL, reducing IgA and Gd–IgA1 levels, which may decrease immune complex deposits in the kidney and reduce proteinuria.	8 mg/kg/d	62	−1.5	−5.9
Sparsentan	Sparsentan is a dual endothelin and angiotensin receptor antagonist that reduces proteinuria by blocking the corresponding pathways.	400 mg/d	42.8	−2.7	−3.8
Atrasentan	Selective endothelin A receptor antagonist	0.75 mg/d	38.1	NA	NA
Iptacopan	Factor B inhibitor of the alternative complement pathway, inhibiting complement‐mediated kidney damage	400 mg/d	38.3	NA	−6.6

From direct B cell depletion strategies to more nuanced modulation of B cell activation pathways, these emerging therapies offer hope for more effective and potentially safer treatment options for patients with IgAN. The ongoing clinical trials and basic science research in this area promise to shed further light on the most effective ways to target B cells in IgAN.

### 6.3 Complement Inhibitors

**FIGURE 4:**
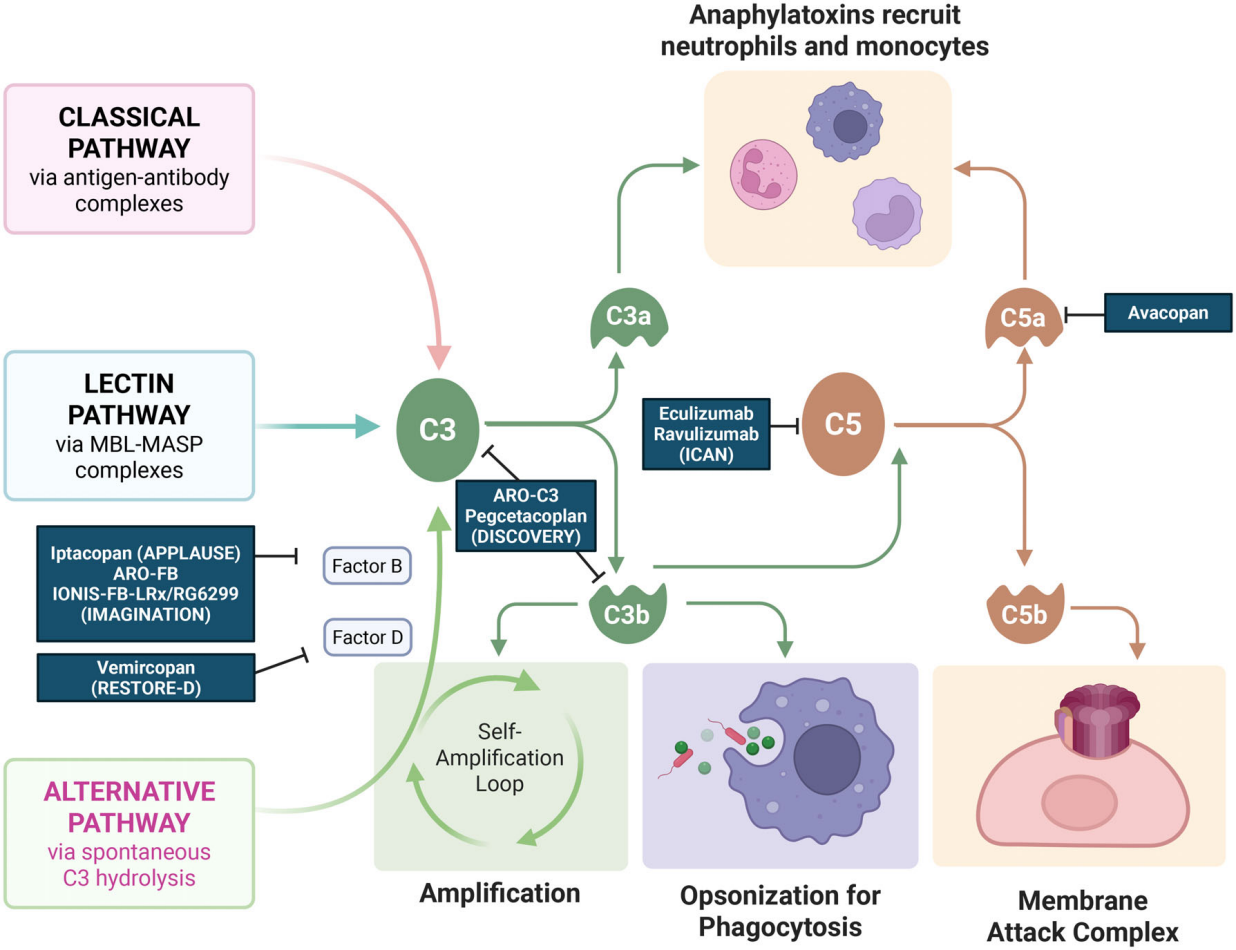
Complement activation pathways and therapeutic targets in IgA nephropathy. It illustrates the dysregulated complement cascade in IgAN, highlighting the alternative and lectin pathways as primary drivers of glomerular injury. Pathogenic immune complexes containing Gd–IgA1 deposit in the mesangium, triggering robust activation of the alternative pathway through three interconnected mechanisms: Factor B‐dependent C3 convertase (C3bBb) assembly, amplified by properdin‐mediated stabilization and impaired regulation due to genetic variants in complement factor H‐related proteins (CFHR1/CFHR5). This creates a self‐sustaining amplification loop that overwhelms endogenous regulatory mechanisms. Downstream effector generation: Cleavage of C3 and C5 releases potent anaphylatoxins (C3a/C5a), which recruit proinflammatory macrophages and neutrophils, exacerbating oxidative stress and cytokine production (IL‐6, TNF‐α). Concurrently, membrane attack complex (MAC/C5b‐9) formation induces direct podocyte lysis and endothelial injury. C5a receptor (C5aR1) signaling, which sustains a profibrotic microenvironment via TGF‐β activation and macrophage chemotaxis. Therapeutic interventions specifically target alternative pathway nodes: Iptacopan inhibits factor B, blocking C3 convertase formation and interrupting the amplification cycle at its origin. Avacopan antagonizes C5aR1, mitigating chemotaxis and cytokine release driven by C5a. Ravulizumab prevents C5 cleavage, inhibiting MAC assembly and terminal pathway‐mediated cytotoxicity. Biomarkers of alternative pathway activation (urinary C5b‐9, glomerular C3d) correlate with histologic severity and predict treatment response, enabling personalized therapy. Terminal pathway inhibition carries infection risks (e.g., Neisseria), necessitating prophylactic strategies. This framework underscores the alternative pathway's centrality as a diagnostic biomarker and therapeutic target in IgAN, with lectin pathway involvement noted as a secondary contributor in select patient subsets.

Complement inhibitors have emerged as a promising therapeutic approach in IgAN, driven by growing evidence of complement activation's role in disease pathogenesis [288, 289, 290, 291, 292, 293] (Figure 4). Glomerular deposition of complement components, particularly C3, is a hallmark of IgAN and correlates with disease severity and progression. Recent mechanistic studies have elucidated mechanisms linking glomerular IgA deposition to complement activation and subsequent inflammation, focusing on the alternative and lectin pathways [289, 294, 295].

The AP has been implicated through increased levels of factor H‐related proteins (CFHRs) in IgAN patients. CFHR1 and CFHR5 compete with factor H for C3b binding, potentially leading to dysregulation and increased complement activation. The lectin pathway's involvement is evidenced by glomerular deposition of MBL and MBL‐associated serine proteases (MASPs) in IgAN kidney biopsies, suggesting activation by aberrantly glycosylated IgA1 molecules. Genetic studies have further supported complement's role, identifying associations between IgAN susceptibility and variants in complement‐encoding genes.

Complement inhibitors can be broadly categorized based on their targets within the complement cascade, including inhibitors of the AP, the lectin pathway (LP), and the terminal pathway (TP). Iptacopan, a factor B inhibitor, prevents C3 convertase formation, reducing overall complement activation [185, 289]. In the phase 3 APPLAUSE‐IgAN study, iptacopan demonstrated a 38.3% reduction in proteinuria compared with placebo at 9 months (*p* < 0.0001) [185], with consistent efficacy across key subgroups and good tolerability. Narsoplimab, targeting MASP‐2 in the lectin pathway, showed promise in early‐phase trials [296, 297], with a median reduction of 61.4% in 24‐h urine protein excretion during a dose‐extension phase. TP inhibitors, such as avacopan, inhibit C5a receptor signaling to mitigate MAC‐mediated injury, have also shown potential in small pilot studies.

The safety profile of complement inhibitors in IgAN appears to be generally favorable, with most studies reporting good tolerability. However, as with any immunomodulatory therapy, there are potential risks that need to be carefully considered. These may include an increased susceptibility to certain infections, particularly those caused by encapsulated bacteria (*Streptococcus pneumoniae*, *Neisseria meningitidis*, *Haemophilus influenzae*), due to the important role of complement in host defense. Prophylactic vaccination and antimicrobial coverage are essential but may not fully mitigate this risk, especially in endemic regions. Long‐term safety data from larger cohorts of patients with IgAN treated with complement inhibitors are still needed to fully characterize the risk–benefit profile of these therapies. The unanswered questions include: chronic immunosuppression and secondary infection vulnerability, potential for compensatory pathway activation (e.g., classical pathway overriding alternative blockade), and renal and hepatic toxicity with extended use, observed in trials of TP inhibitors.

As our understanding of the role of complement in IgAN pathogenesis continues to evolve, and as more clinical data become available, these targeted therapies have the potential to significantly improve outcomes for patients. The coming years will be critical in determining how best to integrate complement inhibitors into clinical practice, with the ultimate goal of reducing disease progression and improving quality of life.

### 6.4 Cellular Therapies

Cellular therapies have emerged as a promising frontier in the treatment of autoimmune disorders [298, 299, 300, 301, 302, 303, 304], including IgAN. These innovative approaches, encompassing stem cell therapies, CAR‐T cells, CAR‐natural killer (NK) cells, and CAR‐macrophages, represent a paradigm shift in addressing complex immune‐mediated conditions. While still in infancy, the potential application of these therapies to IgAN offers exciting possibilities for this challenging kidney disease (Table 5).

**TABLE 5:** Comparison of cellular therapies for IgA nephropathy.

Therapy	Target(s)	Mechanism	Advantages	Challenges
CAR‐T cells	CD19, CD20, CD38, BCMA, platelet‐derived growth factor receptor β (PDGFRβ)	Genetically engineered T‐cells expressing chimeric antigen receptors (CARs) targeting B‐cell/plasma cell surface antigens and targeting surface antigen of extracellular matrix (ECM)‐producing cells in the kidney; induces direct cytotoxicity via perforin/granzyme release	Durable remission after single infusion; high specificity for pathogenic cells	CRS (15–20%); neurotoxicity; prolonged B‐cell aplasia; high cost ($500K+); 3–4‐week manufacturing
CAR‐NK cells	CD19, CD38, mesangial antigens (e.g., β2‐spectrin)	Engineered NK cells with CAR specificity + innate cytotoxicity; “off‐the‐shelf” allogeneic potential	Lower CRS risk; shorter lifespan reduces long‐term immunosuppression concerns	Limited persistence (may require repeat dosing); poor renal homing; scalability challenges
CAR‐macrophages	Mesangial antigens (e.g., β2‐spectrin), IgA deposits	CAR‐engineered macrophages phagocytose immune complexes or promote anti‐inflammatory (M2) polarization; modulates local renal immune milieu	Tissue repair capability; potential for resolving glomerular IgA deposits	Scalable manufacturing hurdles; tumorigenicity risks; limited clinical data in IgAN
Mesenchymal stem cells (MSCs)	N/A (immunomodulation)	Paracrine signaling (TGF‐β, IL‐10); promotes T‐reg expansion; inhibits B‐cell hyperactivity; antifibrotic effects	Favorable safety profile; reduces autoantibody production	Poor renal homing (<10% engraftment); variable potency; requires repeated dosing
Enhanced MSC strategies	‐ CXCR4 (homing) ‐ Anti‐inflammatory transgenes (e.g., IL‐10)	Engineered MSCs with homing receptors or therapeutic transgenes to enhance renal targeting and immunomodulation	Improved tissue‐specific delivery; synergistic effects with CAR therapies	Complexity in manufacturing; genotoxicity risks; regulatory hurdles for genetically modified products
Hematopoietic stem cells (HSCs)	Immune reset	Reconstitutes immune system after myeloablation; eliminates autoreactive B‐cell clones	Potential for long‐term remission; curative potential	High toxicity (infections, graft‐versus‐host disease (GVHD)); limited IgAN‐specific data; ethical/logistical constraints

#### 6.4.1 Mesenchymal Stem Cell Therapy

Mesenchymal stem cell (MSC) therapy has shown considerable promise in treating autoimmune diseases due to its immunomodulatory properties. In lupus nephritis, clinical trials have demonstrated MSCs' ability to reduce disease activity, improve renal function, and decrease the need for immunosuppressive medications [305, 306, 307, 308]. The mechanism involves secretion of anti‐inflammatory cytokines, promotion of regulatory T‐cell function, and inhibition of B‐cell and T‐cell proliferation. The success of MSC therapy in lupus nephritis provides a compelling rationale for exploring its potential in IgAN. Both conditions share some common immunological features, including dysregulated B‐cell function and the production of pathogenic antibodies. However, organ‐specific delivery remains a hurdle: only 5–10% of intravenously administered MSCs home to inflamed kidneys. Strategies like intra‐arterial infusion or MSC engineering with kidney‐homing ligands (e.g., CXCR4 overexpression) are under investigation to enhance renal targeting.

#### 6.4.2 CAR‐T Cell Therapy

CAR‐T cell therapy, which has revolutionized the treatment of certain hematological malignancies, is now being investigated for autoimmune diseases. This approach involves genetically modifying T‐cells to express CARs that recognize specific antigens associated with the disease. In the context of autoimmune diseases, CAR‐T cells could be engineered to target autoreactive B‐cells or plasma cells responsible for producing pathogenic antibodies. Early studies in lupus models have shown promising results, with CAR‐T cells targeting CD19 leading to B‐cell depletion and amelioration of disease symptoms [302, 309, 310, 311, 312, 313, 314, 315]. Adapting CAR‐T cell therapy for IgAN presents both exciting possibilities and significant challenges. The key would be to identify an appropriate target antigen specific to the pathogenic cells involved in IgAN. Potential targets could include surface markers on the B‐cells or plasma cells producing galactose‐deficient IgA1, or even the aberrant IgA1 molecule itself. However, the risk of off‐target effects and the potential for long‐term B‐cell depletion must be carefully considered, given the important role of IgA in mucosal immunity. For IgAN, potential targets include: Gd–IgA1‐producing plasmablasts (via CD38/BCMA) and autoreactive B‐cell clones (via CD19/CD20). Early‐phase trials in autoimmune conditions show sustained remission after a single infusion, but safety concerns persist. Cytokine release syndrome (CRS) occurs in 15–20% of patients, and prolonged B‐cell depletion increases infection risk. Additionally, manufacturing complexity (3–4 weeks for autologous product generation) and costs (≥$500,000 per treatment) limit accessibility.

#### 6.4.3 CAR‐NK and CAR‐Macrophage Approaches

CAR‐NK cells offer another promising avenue for cellular therapy in autoimmune diseases and potentially IgAN. NK cells are innate immune cells with potent cytotoxic capabilities [316, 317]. CAR‐NK cells combine the natural cytotoxicity of NK cells with the specificity of CAR technology. This approach may offer advantages over CAR‐T cells, including a potentially better safety profile due to the limited lifespan of NK cells and their reduced propensity to cause CRS. Their shorter lifespan reduces long‐term immunosuppression concerns, but limited persistence may necessitate repeat dosing.

The concept of CAR‐macrophages represents a recent development in cellular therapies [318]. Given macrophages' crucial roles in inflammation, tissue repair, and immune regulation, CAR‐macrophages could be engineered to target specific cells or tissues involved in IgAN pathogenesis. This approach could modulate the local immune environment in the kidney, potentially promoting an anti‐inflammatory state and facilitating tissue repair and IgA deposit resolution. CAR‐macrophages can be engineered to phagocytose immune complexes or promote anti‐inflammatory polarization (M2 phenotype). Preclinical data suggest efficacy in resolving glomerular immune complex deposits, but scalable manufacturing and tumorigenicity risks require further validation.

#### 6.4.4 Critical Barriers to Clinical Implementation

While cellular therapies represent a transformative frontier for IgAN, significant translational barriers must be overcome before clinical integration. Foremost is the organ‐specific targeting challenge: intravenous delivery achieves <5% renal parenchymal engraftment due to inefficient homing. This necessitates advanced strategies such as intra‐arterial infusion or engineering homing receptors (e.g., CCR2 for inflamed glomeruli or β2‐spectrin‐specific CARs to enhance renal localization. Long‐term safety concerns include risks of persistent B‐cell aplasia (particularly with CD19‐targeted CAR‐T cells), which may require Ig replacement, and genomic instability from viral vectors. Durability limitations are pronounced in short‐lived CAR‐NK cells, often mandating repeated dosing that escalates cumulative toxicity risks.

Manufacturing complexity presents another major hurdle: autologous CAR‐T production requires specialized good manufacturing practice facilities, elevating costs to $400,000–$1.5 M per patient. Allogeneic “off‐the‐shelf” products, while potentially reducing costs, carry graft‐versus‐host disease risks. Regulatory frameworks for nononcologic cell therapies remain underdeveloped, lacking standardized efficacy benchmarks for IgAN‐specific endpoints like proteinuria reduction or eGFR stability. Additionally, the heterogeneous pathogenesis of IgAN demands combination strategies—such as MSC‐primed immunotolerant microenvironments paired with CAR‐T/NK cells targeting Gd–IgA1‐producing plasmablasts—which introduce further clinical trial design complexities.

The organ‐specific nature of IgAN (primarily manifesting in the kidneys) underscores the need for precise targeting. Strategies like kidney‐homing peptides or localized administration techniques require development to improve efficacy. Long‐term safety, durability of treatment effects, and potential need for repeated interventions must be rigorously evaluated. Given the complexity of IgAN pathogenesis, combination approaches may be reasonable—for example, integrating MSC therapy to modulate systemic immune environments with CAR‐T/NK cytotoxicity against pathogenic B‐cell clones. Such synergies could simultaneously dampen inflammation and eliminate disease‐driving cell populations.

Regulatory and manufacturing challenges include standardizing production protocols and advancing allogeneic platforms to improve accessibility. International consortia (e.g., KDIGO) are now defining IgAN‐specific efficacy endpoints to guide regulatory pathways. Future directions may include developing kidney‐targeted CARs against mesangial‐specific antigens (e.g., β2‐spectrin) to enhance renal specificity while sparing extrarenal tissues, cost‐reduction tactics like automated bioreactors (potentially lowering expenses by 60–70%), and leveraging deeper pathogenesis insights to move beyond symptom management toward disease modification. As cellular technologies advance, these therapies hold promises for fundamentally altering IgAN progression, offering hope for improved outcomes.

### 6.5 Relevant Preclinical Animal Experiments and Clinical Trials

The integration of preclinical animal models and clinical trials has been pivotal in advancing our understanding of IgAN pathogenesis and accelerating therapeutic development. Animal models recapitulate key aspects of human IgAN, including mesangial IgA deposition, hematuria, proteinuria, and progressive renal injury. These models have elucidated critical pathophysiological mechanisms, such as aberrant IgA glycosylation, immune complex formation, complement activation, and mucosal immune dysregulation. Concurrently, insights from preclinical studies have directly informed the design of clinical trials targeting these pathways, resulting in a rapidly evolving therapeutic landscape.

#### 6.5.1 Preclinical Animal Models: Mechanistic Insights and Contributions

Several animal models have been instrumental in dissecting IgAN pathogenesis [6, 9, 11, 14, 15, 17, 319, 320, 321, 322]. The ddY mouse model, particularly its subgroup the grouped ddY (gddY) strain, spontaneously develops IgAN‐like features, including mesangial IgA–IgG–C3 deposition, proteinuria, and glomerulosclerosis [58, 94, 112, 140, 258, 323, 324, 325, 326]. This model has been foundational for studying the role of TLRs. Activation of TLR9 by microbial DNA analogs exacerbates IgAN in gddY mice, driving the production of Gd–IgA1 and immune complexes via the MyD88–NF‐κB pathway. Subsequent work demonstrated that HCQ, a TLR7/9 inhibitor, suppresses aberrant IgA glycosylation and renal injury in this model [58, 112], providing preclinical rationale for its clinical evaluation in IgAN patients. The gddY model also illuminated the role of APRIL (a proliferation‐inducing ligand). APRIL overexpression in gddY mice promotes pathogenic IgA synthesis, while anti‐APRIL antibodies reduce serum Gd–IgA1, mesangial deposition, and proteinuria [326]. This directly supported the development of anti‐APRIL therapies now in clinical trials. Complementary models, such as the β‐1,4‐galactosyltransferase‐I‐deficient mouse [327], highlighted the significance of aberrant IgA glycosylation, while humanized models (e.g., α1KICD89Tg mice expressing human IgA1 and CD89) confirmed the role of Fcα receptor‐mediated mesangial injury [328, 329, 330]. ET‐1 signaling was identified as a key mediator of podocyte injury and proteinuria, leading to preclinical testing of ERAs like sparsentan, which preserved podocyte integrity and reduced glomerulosclerosis [331]. Using bacterial lipocalin‐type prostaglandin D synthase (LCWE) to induce immune complexes, it demonstrated that complement activation (via the AP) is essential for glomerular deposition and injury. This model validated the “multi‐hit” hypothesis, showing that Gd–IgA1–IgG complexes trigger endothelial glycocalyx disruption, facilitating mesangial migration and inflammation [332]. These models collectively underscore the importance of mucosal immune dysregulation (e.g., TLR/BAFF/APRIL pathways), nephritogenic immune complex formation (Gd–IgA1–autoantibody interactions), and downstream effectors (complement, ET, and inflammatory cytokines).

Animal models have not only identified therapeutic targets but also enabled combination therapy testing. For example, gddY studies showed that SGLT2 inhibitors (e.g., dapagliflozin) synergize with ET antagonists to mitigate proteinuria and glomerular hyperfiltration, prompting trials like NCT05856760 (SPARTACUS) evaluating sparsentan with SGLT2 inhibitors.

Recent models have also emphasized disease heterogeneity. Genetic analyses of gddY mice revealed subphenotypes with distinct TLR/APRIL dysregulation, suggesting personalized therapeutic approaches. This approach is consistent with current clinical strategies to stratify patients using biomarkers (such as serum Gd–IgA1 and APRIL levels) in ongoing trials, including NCT05732402 (RUBY‐3).

Preclinical models remain indispensable for deconstructing IgAN pathogenesis and validating therapeutic targets. Their translation to clinical trials has yielded multiple targeted therapies, with three agents (nefecon, sparsentan, iptacopan) approved and dozens in late‐phase testing. Future directions include leveraging models to optimize combination regimens, identify predictive biomarkers, and address advanced‐disease populations.

#### 6.5.2 Human Clinical Trials: Translation of Preclinical Insights

The mechanistic insights derived from animal models have directly informed an unprecedented expansion in clinical trial activity for IgAN, fundamentally transforming the therapeutic landscape. The translation from bench to bedside has been particularly successful, with multiple pathways identified in preclinical studies now being targeted in phases 2 and 3 clinical trials. This robust pipeline represents the culmination of decades of animal model research that elucidated key pathogenic mechanisms, providing the scientific rationale for mechanism‐based therapeutic interventions.

Mucosal‐targeted therapies represent the first successful translation from animal models to clinical practice. The observation that gddY mice maintained in germ‐free conditions did not develop IgAN, coupled with the demonstration that GALT plays a central role in aberrant IgA production [333], provided the foundation for developing targeted‐release budesonide (nefecon). This approach specifically targets IgA synthesis in Peyer's patches of the terminal ileum, where pathogenic IgA1 is predominantly produced. The NefIgArd phase 3 trial demonstrated that nefecon significantly reduced proteinuria and slowed eGFR decline, leading to regulatory approval as the first disease‐specific therapy for IgAN [186]. The success of this gut‐targeted approach validated decades of animal research demonstrating the central role of mucosal immunity in IgAN pathogenesis.

B‐cell modulation strategies have emerged as particularly promising therapeutic approaches based on extensive preclinical validation. The discovery that APRIL overexpression in gddY mice promotes pathogenic IgA synthesis, while anti‐APRIL antibodies reduce serum Gd–IgA1 and mesangial deposition, provided compelling rationale for clinical development [112]. Sibeprenlimab, a humanized anti‐APRIL monoclonal antibody, demonstrated remarkable efficacy in the phase 2 ENVISION trial, with 47%–62% proteinuria reduction across different dose levels and stabilization of eGFR compared with progressive decline in the placebo group [282, 283]. The VISIONARY phase 3 trial has completed enrollment and will provide definitive evidence for this mechanism. Similarly, zigakibart (BION‐1301), another anti‐APRIL antibody [334], showed 53% proteinuria reduction in phase 1/2 studies and is advancing to the BEYOND phase 3 trial.

The success of dual APRIL/BAFF inhibition in preclinical models led to clinical testing of TACI‐Fc fusion proteins. Atacicept demonstrated dose‐dependent proteinuria reduction and kidney function stabilization in the phase 2b ORIGIN trial, with concurrent reductions in serum Gd–IgA1 levels confirming target engagement [335, 336]. The ORIGIN 3 phase 3 trial is currently recruiting patients worldwide. Telitacicept, approved for systemic lupus erythematosus in China, showed similar efficacy in IgAN with significant reductions in plasma Gd–IgA1 and IgG–IgA immune complexes, providing direct confirmation of the pathogenic mechanisms identified in animal models [285, 337]. Povetacicept, featuring a reengineered TACI domain with enhanced potency, demonstrated 53.5% proteinuria reduction in early‐phase studies, with the RAINIER phase 3 trial in development [64].

Complement inhibition represents another successful translation from preclinical research. The demonstration in LCWE‐complement models that AP activation is essential for glomerular deposition and injury provided strong rationale for targeting this cascade [332]. Iptacopan, an oral factor B inhibitor, achieved 43.8% proteinuria reduction in the phase 2 trial and has received US FDA accelerated approval based on interim phase 3 results from the APPLAUSE‐IgAN study [20, 91, 289, 338]. The antisense oligonucleotide RO7434656, which reduces hepatic factor B production, demonstrated 45% proteinuria reduction in phase 2 studies and is advancing to the IMAGINATION phase 3 trial. Terminal complement inhibition with ravulizumab showed 29.8% proteinuria reduction in the SANCTUARY trial, leading to the initiation of the ICAN phase 3 study [90, 195].

ERA emerged from animal studies demonstrating that ET‐1 signaling mediates podocyte injury and proteinuria in IgAN models. The gddY mouse studies showing that ET antagonists preserve podocyte integrity and reduce glomerulosclerosis provided direct preclinical validation for this approach [331, 339, 340]. Sparsentan, a dual ET/angiotensin receptor antagonist, demonstrated superior eGFR preservation compared with irbesartan in the rigorous PROTECT phase 3 trial, leading to regulatory approval. The mechanism‐of‐action studies in animal models, showing protection of glomerular glycocalyx and prevention of immune complex migration into the mesangium, were directly confirmed by biomarker studies in the clinical trial. Atrasentan, a selective ETA receptor antagonist [200, 202, 234, 341], achieved 36.1% proteinuria reduction in the ALIGN trial interim analysis.

Plasma cell depletion strategies, based on observations that rituximab failed to reduce Gd–IgA1 levels due to its inability to target tissue‐resident plasma cells, led to development of anti‐CD38 therapies. Felzartamab demonstrated sustained reductions in proteinuria and Gd–IgA1 levels extending beyond 10 months after dosing completion in the IGNAZ trial, confirming that plasma cells are indeed critical sources of pathogenic IgA1 [342, 343].

The comprehensive clinical trial portfolio shown in Table 6 illustrates the breadth of mechanism‐based approaches now under investigation. Three therapies (nefecon, sparsentan, iptacopan) have achieved regulatory approval, with multiple others in late‐phase development. The consistency between preclinical predictions and clinical outcomes validates the utility of animal models in IgAN research and demonstrates the power of mechanism‐based drug development. Current trials are exploring combination approaches, building on animal studies showing synergistic effects between different therapeutic targets, with the goal of achieving more profound and durable treatment responses for patients with this progressive kidney disease.

**TABLE 6:** Important ongoing clinical trials for IgA nephropathy.

Drug name	Target/mechanism	Type	Phase	Trial name	NCT number	Status	Key findings/outcomes	Geographic focus
**B‐cell targeted therapies**								
Sibeprenlimab (VIS649)	APRIL inhibition	Humanized mAb	Phase 3	VISIONARY	NCT05248646	Active, not recruiting	Phase 2: 47–62% proteinuria reduction; eGFR stabilization	Global
Zigakibart (BION‐1301)	APRIL inhibition	Humanized mAb	Phase 3	BEYOND	NCT05852938	Recruiting	Phase 1/2: 53% proteinuria reduction at 52 weeks	Global
Atacicept	APRIL + BAFF inhibition	TACI‐Fc fusion protein	Phase 3	ORIGIN 3	NCT04716231	Recruiting	Phase 2b: proteinuria reduction and kidney function stabilization	Global
Telitacicept	APRIL + BAFF inhibition	TACI‐Fc fusion protein	Phase 3	—	NCT05799287	Recruiting	Phase 2: dose‐dependent proteinuria reduction	China
Povetacicept	APRIL + BAFF inhibition	TACI‐Fc fusion protein	Phase 2	RUBY‐3	NCT05732402	Recruiting	Interim: 53.5% proteinuria reduction; 60% Gd–IgA1 reduction	Global
Felzartamab	CD38 inhibition	Anti‐CD38 mAb	Phase 2	IGNAZ	NCT05065970	Active, not recruiting	Sustained proteinuria and Gd–IgA1 reduction >10 months	Global
**Complement inhibitors**								
Iptacopan (LNP023)	Factor B inhibition	Small molecule	Phase 3	APPLAUSE‐IgAN	NCT04578834	Active, not recruiting	43.8% proteinuria reduction; US FDA approved	Global
RO7434656	Factor B inhibition	Antisense oligonucleotide	Phase 3	IMAGINATION	NCT05797610	Recruiting	Phase 2: 45% proteinuria reduction	Global
Ravulizumab	C5 inhibition	Anti‐C5 mAb	Phase 3	ICAN	NCT06291376	Recruiting	Phase 2: 29.8% proteinuria reduction	Global
Cemdisiran	C5 inhibition	siRNA	Phase 2	—	NCT03841448	Completed	37.4% proteinuria reduction	Global
**Endothelin receptor antagonists**								
Sparsentan	Dual ETA/AT1R antagonist	Small molecule	Approved	PROTECT	NCT03762850	Completed	Superior eGFR preservation vs. irbesartan; US FDA/European Medicines Agency (EMA) approved	Global
Atrasentan	ETA receptor antagonist	Small molecule	Phase 3	ALIGN	NCT04573478	Active, not recruiting	Interim: 36.1% proteinuria reduction	Global
**Combination trials**								
Sparsentan + SGLT2i	Dual mechanism	Small molecules	Phase 3	SPARTACUS	NCT05856760	Recruiting	Testing synergistic effects	Global
**Mucosal targeting**								
Nefecon	Gut‐targeted corticosteroid	Targeted‐release budesonide	Approved	NefIgArd	NCT03643965	Completed	US FDA/EMA approved; 5.05 mL/min/1.73 m^2^ eGFR benefit	Global

## 7 Factors Influencing Treatment Initiation

**FIGURE 5:**
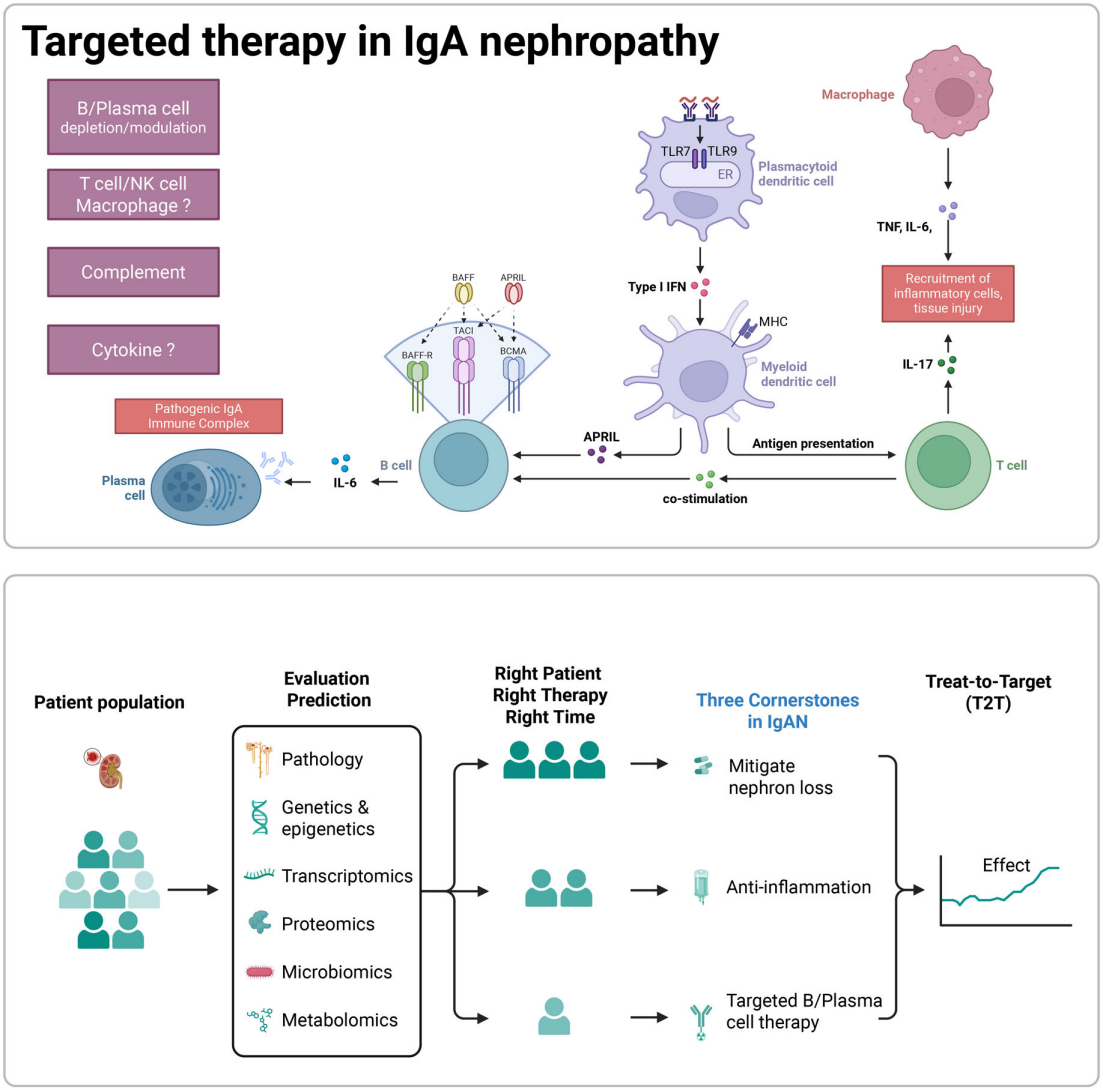
Integrated treatment initiation and treat‐to‐target strategy in IgA nephropathy. It delineates a personalized framework for initiating targeted therapies in IgAN, synthesizing clinical, histological, and biomarker parameters to optimize therapeutic intervention timing. The algorithm prioritizes early intervention for high‐risk patients while avoiding overtreatment in indolent cases. Key clinical triggers include persistent proteinuria exceeding 0.5 g/day despite ≥3 months of optimized supportive care (maximized RASi, blood pressure control <130/80 mmHg, and lifestyle modifications), a declining estimated glomerular filtration rate (eGFR) trajectory (>2 mL/min/1.73 m^2^/year), and persistent microhematuria (>20 red blood cells per high‐power field). Histological risk stratification via the Oxford MEST‐C score—specifically active lesions (mesangial hypercellularity [M1], endocapillary proliferation [E1], crescents [C1/2]) or chronic changes (segmental sclerosis [S1], tubulointerstitial fibrosis [T1/2])—further refines therapeutic urgency. Biomarkers such as elevated serum Gd–IgA1, urinary soluble CD163, and complement activation products (e.g., urinary C5b‐9) provide mechanistic insights into disease activity but require contextual interpretation due to assay variability and prognostic heterogeneity across populations. Patient‐specific factors—including age, comorbidities (e.g., diabetes, cardiovascular disease), and genetic susceptibility—modulate the risk–benefit calculus, favoring tissue‐targeted agents (e.g., TRF‐budesonide) over systemic immunosuppression in vulnerable subgroups. The paradigm advocates concurrent initiation of immunomodulatory therapies (e.g., APRIL/BAFF inhibitors for elevated serum APRIL, complement inhibitors for C3d‐positive biopsies) and chronic kidney disease management (SGLT2 inhibitors, endothelin antagonists) in high‐risk patients, moving beyond traditional stepwise escalation. Treatment efficacy is guided by a treat‐to‐target (T2T) approach, with 3‐month monitoring intervals during induction to assess remission endpoints: proteinuria reduction to <0.3 g/day, hematuria resolution, blood pressure <130/80 mmHg, and eGFR decline stabilized to <1 mL/min/1.73 m^2^/year. This integrated strategy balances immunological control with preservation of renal reserve, addressing both upstream pathogenesis (mucosal dysregulation, autoantibody production) and downstream fibrotic pathways to mitigate lifelong kidney failure risk.

The poor prognosis associated with IgAN in many patients necessitates a more proactive approach to treatment, particularly given the substantial lifetime risk of kidney failure in high‐risk populations. Recent evidence supports a paradigm shift toward earlier intervention, moving beyond reactive strategies to integrated management that simultaneously targets both immune‐mediated pathogenesis and CKD components [344, 345, 346]. Initiating etiological therapy should be based on a comprehensive assessment of clinical presentation, disease activity, and biomarkers indicative of active renal inflammation. This personalized strategy aims to identify patients most likely to benefit from early intervention while avoiding unnecessary exposure to potentially harmful therapies in those with a more benign course (Figure 5).

Key clinical factors must be evaluated holistically. Disease severity—manifested as persistent proteinuria exceeding 0.5 g/day despite ≥3 months of optimized supportive care (including maximal RASi, blood pressure control, and lifestyle modifications)—remains a critical indicator of progression risk. A declining eGFR trajectory, even in the absence of heavy proteinuria, warrants urgent attention. Histological findings from the Oxford Classification (MEST‐C score) provide essential context: active lesions like mesangial hypercellularity (M1), endocapillary proliferation (E1), crescents (C1/2), or chronic changes such as segmental sclerosis (S1) and tubulointerstitial fibrosis (T1/2) stratify risk and guide therapeutic intensity. Notably, the presence of persistent microhematuria (>20 RBC/HPF) may signal ongoing glomerular inflammation and synergistically worsens prognosis when combined with proteinuria, necessitating consideration for immunosuppressive or targeted immunomodulatory therapies.

Emerging biomarkers [347, 348], while promising, require cautious interpretation. Gd–IgA1 is mechanistically central to IgAN pathogenesis, yet its utility as a standalone clinical biomarker remains controversial [111, 114, 170, 349, 350, 351, 352, 353, 354]. Although elevated serum Gd–IgA1 correlates with disease susceptibility and progression in some cohorts, inconsistencies in its relationship with real‐time disease activity limit its reliability for individual treatment decisions. This controversy stems from unresolved questions about whether Gd–IgA1 is a direct pathogenic driver or merely a marker of systemic immune dysregulation, compounded by methodological variability in assays and lack of standardized thresholds. Other biomarkers—such as urinary soluble CD163 (reflecting macrophage activation), complement activation products (e.g., C5b‐9), and autoantibodies against Gd–IgA1—may offer complementary insights but require further validation for routine clinical use. Thus, biomarker integration should augment—not replace—clinical and histological assessments, particularly in borderline cases.

Patient‐specific factors critically modulate treatment choices. Younger patients face higher cumulative lifetime risks, justifying earlier aggressive intervention to preserve kidney function. Conversely, older individuals or those with significant comorbidities (e.g., diabetes, cardiovascular disease) may prioritize safety, favoring conservative management or targeted therapies with lower toxicity profiles. Genetic and ethnic factors also influence outcomes, with Asian populations often exhibiting more aggressive disease. A transformative shift in therapeutic sequencing is emerging: rather than reserving immunosuppression for “supportive care–resistant” cases, high‐risk patients may benefit from concurrent initiation of etiological agents alongside optimized CKD management. This dual approach addresses both upstream immune dysregulation (e.g., via gut‐immune axis modulators like budesonide or APRIL/BAFF inhibitors) and downstream glomerular hyperfiltration/fibrosis pathways (e.g., SGLT2 inhibitors or ET antagonists) to maximize nephron preservation.

In summary, treatment initiation hinges on dynamic risk stratification: proteinuria >0.5 g/day, eGFR decline, high‐risk histology, and hematuria constitute major triggers. Biomarkers like Gd–IgA1 provide mechanistic insights but lack standalone decisional utility. The evolving landscape—emphasizing early, combination‐based strategies—reflects a move toward personalized, proactive management to achieve complete remission (proteinuria <0.3 g/day, stable eGFR, and hematuria resolution) while mitigating long‐term kidney failure risk.

## 8 Concept of T2T in IgAN

The T2T strategy represents a paradigm shift in chronic disease management, emphasizing systematic treatment adjustments at predefined intervals to achieve specific, clinically meaningful endpoints. This approach has demonstrated superior outcomes compared with conventional care in conditions like diabetes, hypertension, and rheumatoid arthritis through large, randomized trials. While T2T remains conceptually underdeveloped in IgAN, emerging evidence supports its applicability. Current understanding identifies critical therapeutic targets: proteinuria reduction to <0.5 g/day (ideally <0.3 g/day), blood pressure control <130/80 mmHg, stabilization of eGFR decline to <1 mL/min/1.73 m^2^/year, and resolution of persistent hematuria. These targets reflect the multifactorial nature of IgAN progression and will evolve alongside advances in pathogenesis understanding [20, 211].

The timing and sequence of therapeutic interventions are crucial considerations within T2T frameworks. Traditional stepwise escalation—beginning with supportive care (lifestyle modifications and maximally tolerated RAS blockade) before introducing immunosuppression—faces challenges in high‐risk patients. Recent evidence advocates for earlier, integrated intervention targeting both immune‐mediated pathogenesis and CKD components simultaneously. This approach recognizes that delayed immunosuppression in rapidly progressive cases may permit irreversible nephron loss, while isolated supportive care often fails to modify underlying immunological drivers. For example, patients with proteinuria >0.5 g/day despite optimized RAS inhibition and significant hematuria (>20 RBC/HPF) may benefit from concurrent initiation of gut‐targeted immunomodulators (e.g., budesonide) or APRIL/BAFF inhibitors alongside SGLT2 inhibitors or ET antagonists rather than sequential trials of monotherapies.

T2T success necessitates biomarker‐guided personalization. Beyond conventional parameters (proteinuria, eGFR slope), integration of histopathological risk stratification (Oxford MEST‐C scores) and emerging biomarkers (galactose‐deficient IgA1, urinary sCD163, complement activation products) allows dynamic risk assessment. Persistent microhematuria, particularly when coexisting with proteinuria, signals ongoing glomerular inflammation and should trigger therapy escalation independent of proteinuria thresholds. The paradigm also demands regular monitoring intervals (e.g., 3‐month assessments during induction, 6‐month thereafter) to evaluate target attainment and guide therapy adjustments. Future T2T frameworks will likely incorporate molecular targets such as reduction of pathogenic IgA1 levels, modulation of mucosal immune responses, and complement pathway inhibition, enabling precision medicine approaches.

Notably, the T2T model must balance urgency of intervention against therapeutic risks. While aggressive early combination therapy shows promise in preserving kidney function, patient‐specific factors (age, comorbidities, genetic background) modulate risk–benefit calculations. Younger patients with active histological lesions warrant rapid dual‐pathway intervention, whereas older individuals with comorbidities may prioritize safer immunomodulatory agents like targeted‐release budesonide over systemic corticosteroids. This individualized strategy—combining clinical, histological, and biomarker data—represents the cornerstone of effective T2T implementation in IgAN.

## 9 Conclusion and Prospects

The landscape of IgAN management is undergoing profound transformation, driven by advances in molecular pathogenesis understanding and targeted therapeutic development. Historically, treatment relied heavily on proteinuria thresholds and reactive escalation, but emerging evidence supports a paradigm shift toward early, dual‐pathway intervention addressing both immune‐mediated glomerular injury and progressive CKD mechanisms. This integrated approach recognizes that delaying immunomodulation in high‐risk patients risks irreversible nephron loss, while isolated supportive care often fails to modify underlying immunological drivers. The approval of disease‐specific agents—sparsentan (dual ET/angiotensin inhibition), nefecon (targeted mucosal immunomodulation), and iptacopan (complement blockade)—alongside repurposed CKD therapies (e.g., SGLT2 inhibitors) enables synergistic combinations for complete remission.

Critical to this evolution is refined risk stratification beyond proteinuria alone. Histological activity (Oxford MEST‐C scores), persistent microhematuria (>20 RBC/HPF), and biomarkers like galactose‐deficient IgA1 or urinary sCD163 provide nuanced insights into progression risk. For instance, hematuria resolution—spontaneous or treatment‐induced—strongly correlates with renal survival, making it a key therapeutic target alongside proteinuria reduction and eGFR stabilization. This heterogeneity necessitates divergent approaches: for chronic, slowly progressive disease, step‐up therapy with minimal sequential agents avoids overtreatment; in rapidly progressive subgroups, early intensive immunosuppression counters irreversible glomerulosclerosis. While early intervention in high‐risk patients is justified, overtreatment of indolent cases remains a concern.

Looking ahead, three priorities define the next era:

1. Comparative treatment trials evaluating de‐escalated regimens for low‐risk patients and combination therapies for high‐risk subtypes (e.g., APRIL inhibitors with SGLT2i/ET antagonists), measuring not only short‐term proteinuria reduction but also long‐term renal survival and quality of life.
2. Biomarker‐driven algorithms incorporating dynamic risk assessment (e.g., urinary C5b‐9 monitoring during complement blockade) with real‐world evidence tracking risk‐stratified implementation to refine precision dosing.
3. Generating evidence for populations facing therapeutic voids—particularly those historically excluded from trials due to complexity (e.g., rapidly progressive crescentic IgAN, pregnancy, or posttransplant recurrence)—through dedicated registry studies and real‐world data initiatives.

Ultimately, the convergence of molecular insights, targeted therapies, and pragmatic trial designs promises to transform IgAN into a manageable disease with definable remission endpoints. Success requires multidisciplinary collaboration to validate surrogate endpoints (complete remission: proteinuria <0.3 g/day, hematuria resolution, eGFR slope <−1 mL/min/year) and ensure equitable access. Well‐designed trials and real‐world evidence will be essential to balance immunological control with renal reserve preservation, replacing reactive, proteinuria‐centric dogma with proactive, pathophysiology‐driven strategies prioritizing long‐term kidney survival.

## Author Contributions

**Xu‐Jie Zhou**: conceptualization, data curation, writing, and visualization. The author has approved the final manuscript.

## Ethics Statement

The author has nothing to report.

## Conflicts of Interest

The author declares no conflicts of interest.

## Data Availability

The data that support the findings of this study are available from the corresponding author upon reasonable request.
